# Measuring human context fear conditioning and retention after consolidation

**DOI:** 10.1101/lm.053781.123

**Published:** 2023-07

**Authors:** Yanfang Xia, Jelena Wehrli, Samuel Gerster, Marijn Kroes, Maxime Houtekamer, Dominik R. Bach

**Affiliations:** 1 Computational Psychiatry Research, Department of Psychiatry, Psychotherapy, and Psychosomatics, Psychiatric University Hospital Zurich, University of Zurich, 8032 Zurich, Switzerland; 2 Donders Institute for Brain, Cognition, and Behaviour, Radboud University Medical Centre, Nijmegen 6525 GA, the Netherlands; 3 Wellcome Centre for Human Neuroimaging, Max Planck UCL Centre for Computational Psychiatry and Ageing Research, University College London, London WC1 3BG, United Kingdom

## Abstract

Fear conditioning is a laboratory paradigm commonly used to investigate aversive learning and memory. In context fear conditioning, a configuration of elemental cues (conditioned stimulus [CTX]) predicts an aversive event (unconditioned stimulus [US]). To quantify context fear acquisition in humans, previous work has used startle eyeblink responses (SEBRs), skin conductance responses (SCRs), and verbal reports, but different quantification methods have rarely been compared. Moreover, preclinical intervention studies mandate recall tests several days after acquisition, and it is unclear how to induce and measure context fear memory retention over such a time interval. First, we used a semi-immersive virtual reality paradigm. In two experiments (*N* = 23 and *N* = 28), we found successful declarative learning and memory retention over 7 d but no evidence of other conditioned responses. Next, we used a configural fear conditioning paradigm with five static room images as CTXs in two experiments (*N* = 29 and *N* = 24). Besides successful declarative learning and memory retention after 7 d, SCR and pupil dilation in response to CTX onset differentiated CTX^+^/CTX^−^ during acquisition training, and SEBR and pupil dilation differentiated CTX^+^/CTX^−^ during the recall test, with medium to large effect sizes for the most sensitive indices (SEBR: Hedge's *g* = 0.56 and *g* = 0.69; pupil dilation: Hedge's *g* = 0.99 and *g* = 0.88). Our results demonstrate that with a configural learning paradigm, context fear memory retention can be demonstrated over 7 d, and we provide robust and replicable measurement methods to this end.

Learning to predict threat in the environment plays a vital role in evolution and survival across species. A common method to investigate this in the laboratory is Pavlovian fear conditioning (Delgado et al. 2006), also termed threat conditioning (LeDoux 2014). Here, an initially neutral stimulus (conditioned stimulus [CS^+^]) is coupled with a naturally aversive stimulus (unconditioned stimulus [US]) so that the presentation of the CS elicits preparatory responses (conditioned responses [CRs]). In human research, responses evoked by CS^+^ are typically compared with those evoked by a CS^−^, which predicts the absence of the US (Lonsdorf et al. 2017).

When CS^+^ is a distinct elemental cue (so-called cue conditioning), learning is based on synaptic plasticity in the amygdala (LeDoux 2000; Davis and Whalen 2001; Maren 2001). In contrast, CS^+^ can also be a context (CTX^+^) that is comprised of multiple items and their spatial or temporal configuration (for review, see Maren et al. 2013). Encoding the context is thought to involve a conjunctive representation combining the elements with their configuration (Rudy et al. 2004) and requires hippocampal circuitry both in rodents (for review, see Rudy et al. 2004) and in humans (Andreatta et al. 2015a; Baeuchl et al. 2015; Stout et al. 2018, 2019). In standard context conditioning paradigms, US can occur within the context either at unsignaled time points, leading to sustained CR during context presentation, or at predictable time points, often termed signaled context conditioning (Maren et al. 2013). A variant of this paradigm is configural conditioning (Stout et al. 2018, 2019), where the context is only briefly presented and thus the timing of the US is known. We note that while research on hippocampal learning tends to emphasize the configural aspect of context conditioning, other research traditions emphasize the role of US unpredictability (often in the context of investigating sustained defensive responses) and reserve the term “context conditioning” for what we term here “unsignaled context conditioning” (Davis et al. 2010; Andreatta and Pauli 2021). Here, we follow the terminology of Maren et al. (2013) and indicate explicitly whether US is signaled or unsignaled.

To implement context conditioning in nonhuman animals, physically distinct cages are widely used. In humans, three main implementation types can be distinguished. First, distinct physical rooms (Besedovsky et al. 2020) are most similar to nonhuman animal paradigms. Second, different types of static images can be designed to signal a context (Armony and Dolan 2001; Marschner et al. 2008; van Ast et al. 2012; Glenn et al. 2014; Kastner et al. 2015; Kuhn et al. 2016; Stout et al. 2018, 2019; Genheimer et al. 2020). Third, virtual reality (VR) can simulate a physical context both in prerecorded two-dimensional (2D) VR videos (Baas et al. 2004; Grillon et al. 2006; Alvarez et al. 2008; Baas and Heitland 2015) and in three-dimensional (3D) VR, usually semi-immersive without full body movements (Glotzbach et al. 2012; Tröger et al. 2012; Glotzbach-Schoon et al. 2013b, 2015; Mühlberger et al. 2014; Andreatta et al. 2015a,b, 2019, 2020a,b; Kroes et al. 2017; Houtekamer et al. 2020; for reviews, see Glotzbach-Schoon et al. 2013a; Andreatta and Pauli 2021).

In these studies, quantification of human context fear conditioning was mostly based on startle eyeblink responses (SEBRs) (Baas et al. 2004; Grillon et al. 2006; Tröger et al. 2012; Glotzbach-Schoon et al. 2013b, 2015; Glenn et al. 2014; Mühlberger et al. 2014; Baas and Heitland 2015; Kuhn et al. 2016), phasic skin conductance responses (SCRs), and/or tonic skin conductance levels (SCLs) (Armony and Dolan 2001; Alvarez et al. 2008; Marschner et al. 2008; Tröger et al. 2012; Kuhn et al. 2016). Explicit contingency ratings were assessed in most studies as well. Despite a body of work on the differential sensitivity of these CRs in cue conditioning (for review, see Ojala and Bach 2020), a systematic comparison in context conditioning is lacking. Also, it is not known whether and how context memory retention can be measured after a delay of several days, which might be important for research into memory storage as well as for preclinical work in an experimental psychopathology approach (e.g., see Grillon et al. 2004; Bach et al. 2018b, 2019).

Thus, the goal of the present study was (1) to identify a context conditioning paradigm that induces memory retention for several days and (2) to optimize the measurement of the CR during acquisition and retention within such a paradigm. We first considered an unsignaled context conditioning paradigm in 3D VR (adapted from Kroes et al. 2017) in which two rooms with the same furniture but different arrangements serve as CTX^+^ and CTX^−^ (see Fig. 1 for experimental setup; see Table 1 for demography; see also Supplemental Material Tables S1–S3 for experimental details of each trial). While participants were consciously aware of contingencies and retained this declarative memory over 7 d, there was no evidence of learning or memory retention in psychophysiological indices. Thus, we reverted to a signaled context conditioning paradigm, which simplifies psychophysiological analysis, building on a previous configural learning study with static room images (see Fig. 2 for experimental setup; see Table 1 for demography; Stout et al. 2018, 2019). As outcomes, we considered conditioned responses previously used in context conditioning (fear-potentiated startle and skin conductance) as well as pupil size, which is a sensitive measure of cue conditioning (Korn et al. 2017; Bach and Melinscak 2020; Ojala and Bach 2020).

**Figure 1.**
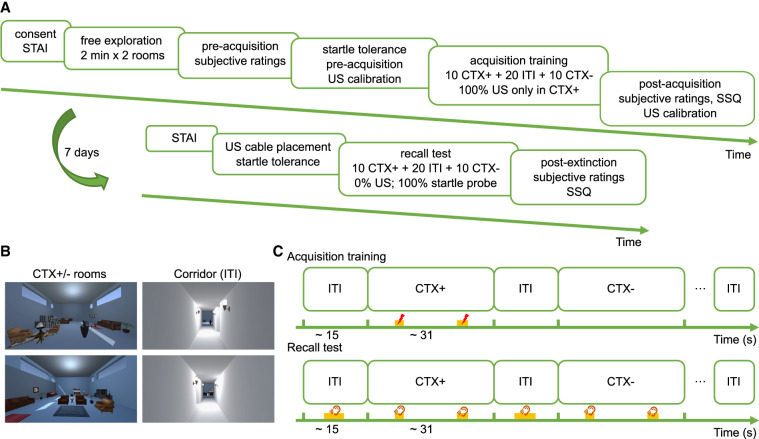
VR task used in experiments 1 and 2. (*A*) Study timeline. (*B*) 2D overview of the two experimental rooms and a white corridor. The two rooms were decorated as living rooms with the same furniture but in different arrangements with a blue background color. The white corridor linked the two rooms (CTX^+^/CTX^−^) and was visited during the intertrial interval (ITI). Assignment of CTX^+^/CTX^−^ to rooms was counterbalanced between participants. Each room image depicted the participants’ view from the entrance. Participants moved passively through the rooms and the corridor in a predefined pseudorandom path. (*C*) Trial-by-trial design of the acquisition training and recall test. In the acquisition training, participants received overall 16 US: two in each of six CTX^+^ trials (one during 6–8 sec after entering the room and one during 21–24 sec) and one in each of the remaining four CTX^+^ trials (6–8 sec after entry). There were no USs in the recall test. To measure fear-potentiated startle eyeblink responses, one startle probe was delivered during each ITI, two were delivered during nine CTX^+^ and CTX^−^ trials, and one was delivered for the remaining one CTX^+^ and CTX^−^ trials. (SSQ) Simulator Sickness Questionnaire, (STAI) State-Trait Anxiety Inventory.

**Figure 2.**
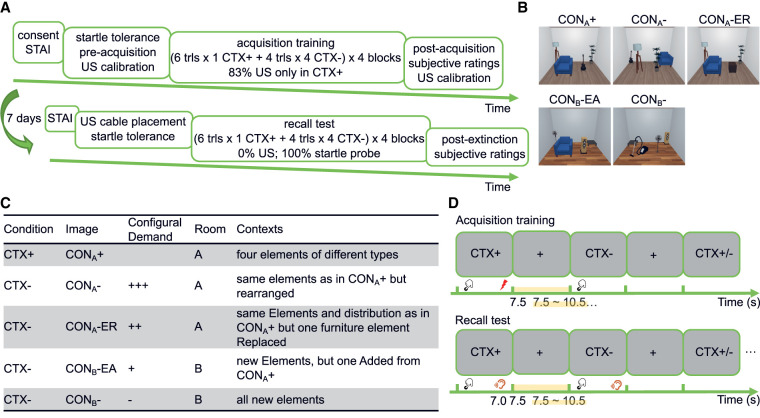
Configural task used in experiments 3 and 4. (*A*) Study timeline. (*B*) Overview of the five static room images used in these experiments. (*C*) Summary table of the design and assignment of the room images (Stout et al. 2018, 2019). For every participant, CTX^+^ was always the CON_A_^+^ image, and all the other images were CTX^−^. Before acquisition training started, all participants were presented an overview of all images on the same screen, where the position of each image was randomized. (*D*) Trial-by-trial design of acquisition training and recall test. In the acquisition training, participants learned the association of rooms and US (electric shock) over 88 trials split into four balanced blocks. In each trial, CTX was presented for 7.5 sec. In 83% of CTX^+^ trials (i.e., five out of six CTX^+^ trials in each block), US was delivered at 7.0 sec after CTX onset and coterminated with CTX. During an ITI with random duration (7.5–10.5 sec), the screen was light gray. Participants were asked to press the “down” arrow key as soon as they figured out the room configuration when a CTX image was presented. In the recall test, no US occurred. In every recall trial (including CTX^−^ trials), a startle probe was delivered at 7.0 sec after trial onset. (STAI) State-Trait Anxiety Inventory.

**Table 1.**
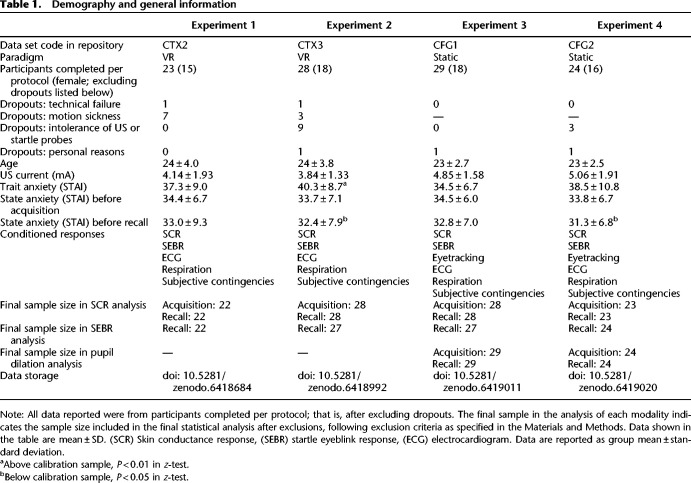
Demography and general information

To determine the most sensitive measure of context fear conditioning and memory retention, we used a calibration approach (Bach et al. 2020). As applied here, this approach assumes that participants learn the CTX–US association and strives to maximize the effect size of the CTX^+^/CTX^−^ difference (i.e., retrodictive validity). Thus, the aim is not to demonstrate in a significant null hypothesis test that participants learn the CTX–US association, but rather to select the measure with the highest effect sizes. To avoid overfitting to a particular sample of limited size, we followed an exploration confirmation strategy with an out-of-sample test. Data from experiments 1 and 3 were explored to maximize effect sizes, and the resulting data analysis algorithms were then confirmed in experiments 2 and 4. Thus, within the exploration sample, multiple comparisons were not a problem, and *P*-values from null hypothesis tests within the exploration samples are only presented as a heuristic guide.

## Results

### Experiments 1 and 2—VR paradigm

#### Induced sickness in VR

We first checked the VR-induced sickness in the participants of experiments 1 and 2. In the Simulator Sickness Questionnaire (SSQ), participants reported higher nausea, oculomotor sickness, and disorientation after both experiments, compared with calibration samples (Supplemental Table S4; Kennedy et al. 1993). This analysis excluded 10 participants who had dropped out due to motion sickness (Table 1).

#### Declarative learning

In both experiments 1 and 2, participants distinguished CTX^+^/CTX^−^ in contingency ratings after the acquisition training and in retrospective ratings after the recall test (Supplemental Fig. S1). This difference was attenuated after the recall test, which was done without reinforcement (Supplemental Tables S5–S8). Participants also reported higher arousal, anxiety, and negative feelings during CTX^+^ compared with CTX^−^, and these differences returned to preacquisition levels after the recall test (Supplemental Fig. S1; Supplemental Tables S5–S8), with the exception of anxiety, which did not return to baseline in experiment 1 only.

#### Conditioned responses in acquisition training

We did not observe any CTX^+^/CTX^−^ difference in SCRs, heart period responses (HPRs), or respiration amplitude responses (RARs) toward trial onset during acquisition training in either experiment. As a positive control to rule out the potential technical failure of skin conductance measurement or nonresponsiveness of the participants to US, SCLs over the entire trial, which are affected by CR as well as US responses in CTX^+^ trials, were higher in CTX^+^ compared with CTX^−^ (*t*_(21)_ = 3.59, *P* = 0.002, *g* = 0.74). This was replicated in experiment 2 (*t*_(27)_ = 3.99, *P* < 0.001, *g* = 0.73).

#### Conditioned responses in the recall test

**Figure 3.**
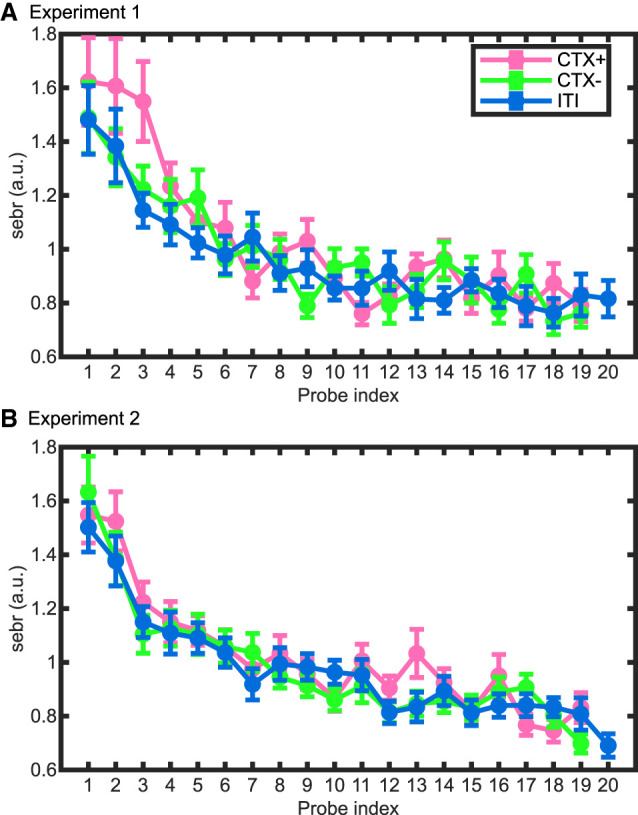
Normalized probe-wise SEBR estimates in experiment 1 (*A*) and experiment 2 (*B*). Linear mixed effect (LME) model analysis on SEBRs did not reveal the difference between CTX^+^ and CTX^−^ but suggested a difference between CTX^+^ and ITI and a main effect of probe index in both experiments. Data represented are group mean ± SEM.

SEBRs in both experiments 1 and 2 revealed a condition effect that was due to a difference between CTX^+^ and ITI, but no CTX^+^/CTX^−^ difference above our threshold of Cohen's *d* = 0.5 (Fig. 3A [for experiment 1], B [for experiment 2]; Supplemental Table S9). There were no above-threshold CTX^+^/CTX^−^ differences in SCLs, SCRs toward trial onset or startle probes, HPRs, or RARs.

### Experiments 3 and 4—configural paradigm

#### Gaze patterns and declarative learning

In both experiments, participants directed their gaze to various furniture items during CTX presentation in acquisition training (Supplemental Figs. S2, S3). Subjectively, participants differentiated the CTX^+^ and CTX^−^ rooms during the acquisition training, and this was attenuated after the recall test, which was done without reinforcement (Supplemental Figs. S4, S5; Supplemental Table S10).

#### Conditioned responses during acquisition

**Figure 4.**
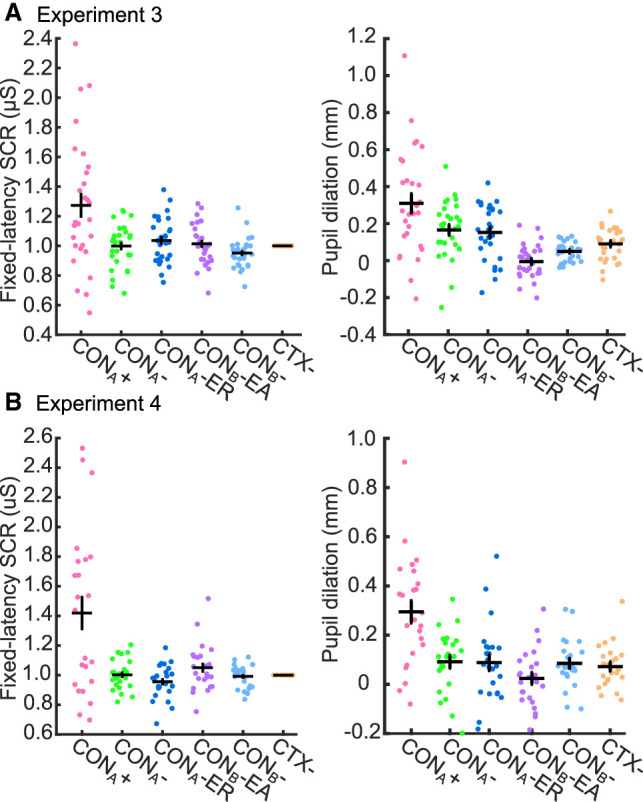
Normalized SCRs to CTX onset (*left*) and pupil dilation (*right*) in acquisition training in experiment 3 (*A*) and experiment 4 (*B*). Only data from nonreinforced trials are included. “CTX^−^” represents averaged data over all four CTX^−^ conditions. Black crosses depict group mean ± SEM.

In experiment 3, we observed a condition effect in a trial-wise linear mixed effect (LME) model analysis (Table 2) for SCRs to CTX onset and pupil dilation in experiment 3 (Fig. 4A). Post-hoc *t*-tests on averaged conditioned responses over all trials in the acquisition training revealed that SCRs (*t*_(27)_ = 3.27, *P* = 0.003, *g* = 0.60) and pupil dilation (*t*_(28)_ = 4.39, *P* < 0.001, *g* = 0.79) were more pronounced for CTX^+^ than for CTX^−^ (Supplemental Table S11). No CTX^+^/CTX^−^ difference was observed in SCRs during CTX, HPRs, or RARs.

**Table 2.**
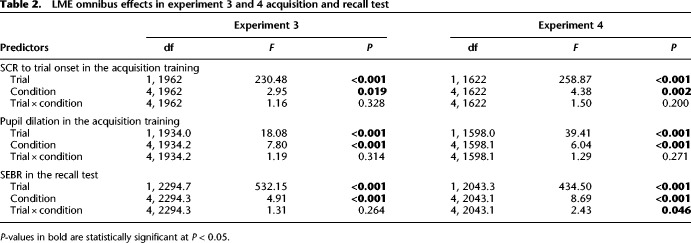
LME omnibus effects in experiment 3 and 4 acquisition and recall test

Experiment 4 confirmed these results for SCRs to CTX onset (post-hoc *t*-test on CTX^+^/CTX^−^ difference: *t*_(22)_ = 3.63, *P* = 0.001, *g* = 0.73) (Table 2; Fig. 4B; Supplemental Table S11) and pupil dilation (*t*_(23)_ = 5.33, *P* < 0.001, *g* = 1.05) (Table 2; Fig. 4B; Supplemental Table S11).

#### Conditioned responses in the recall test

**Figure 5.**
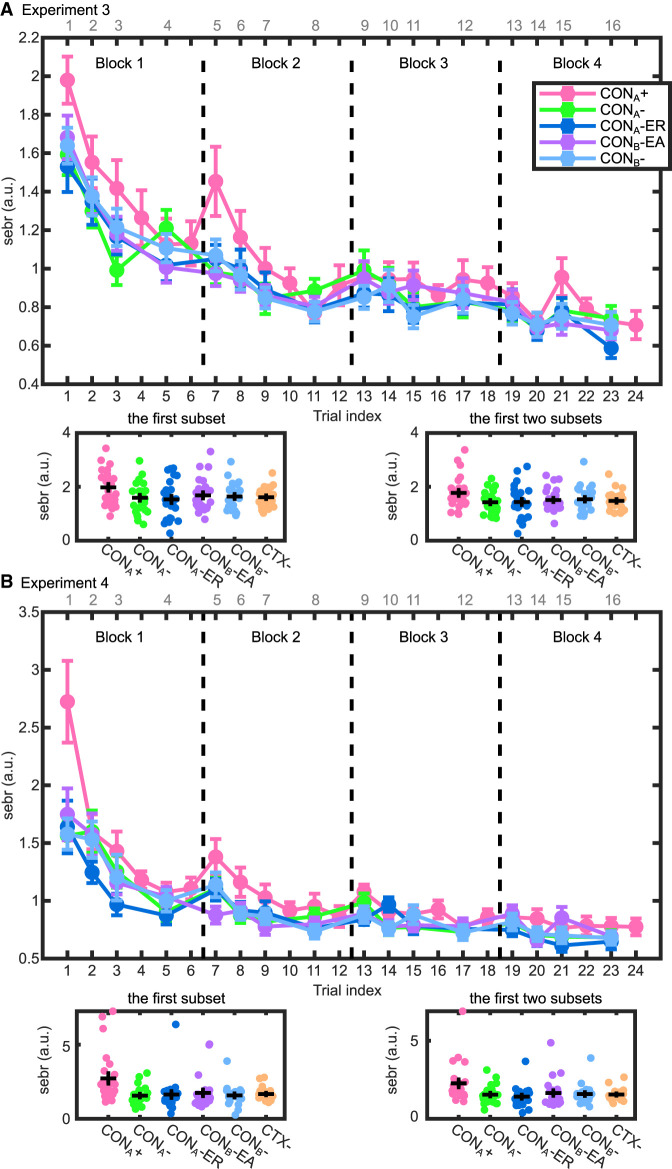
Normalized trial-wise SEBRs during memory recall in experiment 3 (*A*) and experiment 4 (*B*). LME analysis suggested higher SEBRs in CON_A_^+^ than in all CTX^−^ conditions in experiment 3. Post-hoc paired *t*-test suggested the first subset (containing the first trial of each condition) and the average of the first two subsets (containing the first and the second trials of each condition) are the optimal trial numbers to index memory retention. These results were confirmed in experiment 4. Group data are shown as group mean ± SEM. “CTX^−^” is the average over all four CTX^−^ conditions. The *top* of each plot shows the linear trial index of CTX^−^ conditions, and the *bottom* shows the linear trial index of the CTX^+^ condition.

In experiment 3, SEBRs were higher in CTX^+^ than in CTX^−^ trials (Table 2; Fig. 5A; Supplemental Table S12). Since this difference is likely to diminish during the recall test, which was done without reinforcement, we investigated different methods of forming a summary SEBR index for memory retention (Fig. 5A). Averaging the first subset of trials (comprising the first trial of each type) showed the highest effect size to differentiate CTX^+^/CTX^−^ (*t*_(25)_ = 3.00, *P* = 0.006, *g* = 0.56) (Supplemental Table S13); the effect size was attenuated when averaging over the first two subsets. These findings were replicated in experiment 4 (*t*_(23)_ = 3.48, *P* = 0.002, *g* = 0.69) (Table 2; Fig. 5B; Supplemental Tables S12, S14).

In experiment 3, pupil dilation was more pronounced for CTX^+^ than for two of the CTX^−^ rooms (CON_B_^−^ and CON_B_^−^ EA) (Supplemental Fig. S6A; Supplemental Table S15). As a summary index, pupil dilation showed a CTX^+^/CTX^−^ difference that was most pronounced when averaging over the first two subsets (*t*_(28)_ = 5.50, *P* < 0.001, *g* = 0.99) (Supplemental Table S16). For these subsets, each room was different from CTX^+^. These results were replicated in experiment 4 (*t*_(23)_ = 4.46, *P* < 0.001, *g* = 0.88) (Supplemental Fig. S6B; Supplemental Tables S15, S17).

In experiment 3, SCRs during CTX were larger for CTX^+^ than for two of the CTX^−^ rooms (CON_A_^−^ and CON_B_^−^ EA; Supplemental Fig. S7A; Supplemental Table S18). However, there was no overall CTX^+^/CTX^−^ difference in summary indices (Supplemental Table S19). Experiment 4 showed an overall condition effect but a qualitatively different picture in terms of individual condition differences (Supplemental Fig. S7B; Supplemental Tables S18, S20). We did not observe any CTX^+^/CTX^−^ differences during recall in SCRs to CTX onset, HPRs, or RARs.

## Discussion

In this study, we sought to identify the best experimental and analysis strategies in order to induce and measure context fear conditioning and its retention over 7 d. Our primary interest was in paradigms that presumably require hippocampal learning, which is thought to hinge on the necessity to learn about the spatiotemporal configuration of elemental predictor cues.

We first considered a semi-immersive VR paradigm with two configurally different rooms as contextual stimuli and unsignaled US presentation. In two experiments, we found that participants learned—and retained over 7 d—declarative knowledge of the CTX–US contingencies and differential evaluative responses to these stimuli. While they showed a differential unconditioned SCR, no psychophysiological CR index measured in experiments 1 and 2 distinguished CTX^+^/CTX^−^. We next considered a configural learning paradigm with static room images and signaled (temporally predictable) US presentation. Pupil dilation and SCRs toward CTX onset distinguished CTX^+^/CTX^−^ during the acquisition training, and SEBRs as well as pupil dilation distinguished CTX^+^/CTX^−^ in the recall test after 7 d. Among SEBR measures, an index formed over the first trial of each condition turned out to be the most sensitive, with a medium effect size of Hedge's *g* = 0.56 and *g* = 0.69. Among pupil size measures, an index formed over the first two trials of each condition was most sensitive, with Hedge's *g* = 0.99 and *g* = 0.88, respectively.

Previous work using a very similar VR paradigm for acquisition training (Kroes et al. 2017; Houtekamer et al. 2020) consistently demonstrated higher late-phase SEBRs and startle-elicited SCRs in CTX^+^ than in CTX^−^ or ITI, as well as higher SCRs toward trial onset in CTX^+^ compared with CTX^−^ but not with ITI. The main difference between our and the previously reported paradigm is the omission of startle probes in acquisition training, so two of the three previously reported indices were not available. In comparison with other VR studies demonstrating memory retention after overnight consolidation (for review, see Andreatta and Pauli 2021), most of which conducted delayed recall tests after one night of sleep, we used a much longer interval of 7 d between the acquisition training and the recall test. In comparison with a VR study testing the fear memory retention 2 wk after memory acquisition (Andreatta et al. 2017), our SEBR result in the recall test is consistent with their result. Generally, procedural differences in US calibration might lead to overall stronger or weaker learning in different experiments. We did observe potential generalization in the CTX^−^ context compared with the CTX^+^ context in SEBRs, as evident by the contrast versus the ITI room, in line with previous results in VR paradigms (Andreatta et al. 2017; Genheimer et al. 2017; for review, see Andreatta and Pauli 2021) and resembling nonhuman work (Fanselow 1980; Fujinaka et al. 2016; Yu et al. 2021). However, our paradigm was not designed to investigate generalization, which is often assessed by using considerably different rooms as CTX^+^ and CTX^−^ and a third room as a generalization room resembling both the CTX^+^ and CTX^−^ rooms (e.g., Andreatta et al. 2017, 2019, 2020b).

The second paradigm aimed to establish context memory based on configurally different images. We found differential SCRs during the acquisition training, consistent with previous studies (Stout et al. 2018, 2019), as well as differential pupil dilation, with a higher effect size than for SCRs. This provided an additional and robust measure for context memory acquisition in humans and potentially in nonhumans as well (Ojala and Bach 2020).

After 7 d, we observed higher SEBRs and pupil dilation to the CTX^+^ compared with the CTX^−^ in this paradigm. For SEBRs, the effect size was smaller than in previous methodological work on cued fear conditioning (Khemka et al. 2017) but larger than in a human trace fear conditioning study (Wehrli et al. 2022). For pupil size in the recall test, analyses across all trials showed pupil dilation differences only between the CTX^+^ and two of the CTX^−^ conditions, but when considering only the first or first two subsets of trials, all CTX^−^ rooms were different from the CTX^+^ rooms. The resulting index yielded a higher effect size than SEBRs and was replicated in a second independent experiment. It might be useful for the future to investigate pupil size as a memory index in the absence of startle probes, as startle probes could down-regulate the sensitivity of differential fear-potentiated pupil dilation in fear learning (de Haan et al. 2018), and removal of startle probes would simplify the technical requirements of the paradigm and remove the asymmetry of including startle probes in the recall test but not in acquisition training. Pupil size responses have not yet been investigated in the classical cued fear conditioning paradigm over several days (Ojala and Bach 2020) but were reported to not differentiate CSs in human trace fear memory recall after 7 d (Wehrli et al. 2022).

In conclusion, the current study established a human context conditioning paradigm with robust objective measures of memory acquisition and retention over 7 d. The paradigm might be relevant for the investigation of memory retention as well as memory modification studies, which often require memory retention over several days for drug washout (e.g., Grillon et al. 2004; Bach et al. 2018b, 2019).

## Materials and Methods

### Participants

#### Overview

Four independent groups of healthy participants with normal or corrected-to-normal vision were recruited from the general and student population in Zürich (Table 1). All experiments, including the form of taking informed consent, were approved by the governmental research ethics committee (Kantonale Ethikkommission Zürich: KEK-ZH-2013-0118) and were carried out according to the Declaration of Helsinki. After completion of the experiment, participants received monetary compensation based on experiment duration (10 CHF per 30 min). Samples and experiment characteristics are shown in Table 1.

#### Power analysis

In experiments 1 and 3, we were interested in identifying measures in which CTX^+^/CTX^−^ differences are of sufficient size, which we defined a priori as Cohen's *d* = 0.5. There is no established statistical framework to determine the appropriate sample size for this case. Hence, we heuristically computed the sample size required to detect an effect with 80% power (Faul et al. 2007). For experiment 2, we sought to replicate the negative results in experiment 1 in a similarly sized sample. For experiment 4, we based sample size on the smallest hypothesized effect observed in experiment 3. In all experiments, additional participants were included to allow for attrition and quality-related exclusions; hence, the resulting sample sizes vary.

### Experimental procedure

#### Overview

All experiments comprised two visits 7 d apart: acquisition training (visit 1) and recall test (visit 2) with US electrodes attached to the participant but no actual US reinforcement delivered. For all experiments, visit 1 started with the German or English version of the State-Trait Anxiety Inventory (STAI) (Spielberger et al. 1970; Laux et al. 1981), followed by US calibration and startle probe tolerance screening (eight probes with 4-sec ITIs). There were no startle probes during acquisition training, as startle probes might interfere with fear acquisition (Sjouwerman et al. 2016; de Haan et al. 2018). Visit 2 started with the state part of the STAI. Afterward, participants were connected to the US electrode cables, followed by the same type of startle probe as during screening. The recall test included startle probes but not US delivery. Thus, the recall test was formally equivalent to extinction training (Lonsdorf et al. 2017) and might have induced extinction learning (Lacagnina et al. 2019), which we accounted for in our analysis.

Experiments 1 and 2 implemented unsignaled context fear conditioning in semi-immersive 3D VR, modifying a paradigm by Kroes et al. (2017) and Houtekamer et al. (2020). Participants were free to move their heads but could not move their bodies. First, participants familiarized themselves with each of the two rooms for 2 min, during which they could navigate within the VR using hand controllers (Fig. 1A,B). Subsequently, outside the VR, they rated arousal, valence, and anxiety toward each room on a visual analog scale (VAS), followed by a startle probe tolerance screening phase with eight probes and US calibration. Participants were instructed to learn the association of the two rooms with US during fear acquisition, which always commenced in the neutral corridor (Fig. 1C). After acquisition, participants rated arousal, valence, anxiety, US expectancy, and CTX–US contingency, followed by the Simulator Sickness Questionnaire (SSQ) (Kennedy et al. 1993). Finally, US calibration was repeated at the end of visit 1 to investigate changes in US aversiveness.

Experiments 3 and 4 implemented signaled context fear conditioning with static images of different room configurations (Fig. 2B,C), based on Stout et al. (2018, 2019). After US calibration and startle probe tolerance screening (Fig. 2A), the acquisition task (Fig. 2D) commenced with an overview of all room images on one screen, with randomized order of image position. At the end of the acquisition task, participants rated arousal, valence, and CTX–US contingency on a VAS, followed by US calibration.

In experiment 3, the acquisition was followed by a 7.5-min neutral movie and a cue conditioning task (not reported here) with auditory CS and the same US type (with the US electrode applied to a different skin patch) as used in experiment 3.

#### Experiments 1 and 2 (VR task)

We adapted a previously reported VR paradigm by Kroes et al. (2017) and removed color cues differentiating the two rooms, similar to the version reported by Houtekamer et al. (2020). A preliminary test showed only weak explicit contingency learning (and no learning in psychophysiological indices) in the adapted paradigm; hence, we also increased the reinforcement rate to 100% and removed startle probes from the acquisition. The paradigm used here contained two blue living rooms as contextual stimuli (CTX^+^/CTX^−^). A white corridor linked the two rooms and served as the intertrial interval (ITI). Figure 1B shows a 2D still image from each room, from the participants’ view at the entrance of the corresponding room. The two blue rooms were decorated with the same furniture items in different arrangements.

During the acquisition training and recall test, participants were continuously passively guided through the two rooms and the corridor based on a predefined path, while they were free to move and rotate their heads for observation. The tasks started on one side of the corridor, moved to the other side, and entered one of the rooms. They traveled through this room, went back to the corridor, and then moved to a room again. Within each room or corridor, the movement path was pseudorandom so that the place where they received a US or startle probe was always different to avoid potential cue conditioning to nearby furniture items. In total, participants traveled 10 times through each room for ∼31 sec and 20 times through the corridor for ∼15 sec. From the corridor, participants would be guided into the other room (six trials) or back to the same room (four trials) in pseudorandom order (see Supplemental Material Tables S1, S2). The assignment of CTX^+^/CTX^−^ to rooms was counterbalanced between participants. Events (US or startle probes) within the rooms occurred between 6–8 sec and 21–24 sec after entering the room and in the ITI (startle probe) between 5 and 10 sec after entry (Fig. 1C); the actual time point was chosen uniformly at random during these intervals. We note that participants might be able to learn, over time, that US delivery was not uniform over the CS period. However, given the limited number of learning trials and their prior expectation of a uniform distribution, it is unlikely that they would precisely learn the exact boundaries of these intervals. In acquisition training, participants received two USs in each of six CTX^+^ trials and one US in each of the remaining four CTX^+^ trials, and no event occurred during CTX^−^ or ITI visits. In total, participants received 16 USs (Supplemental Tables S1, S2). In the recall test, the US electrode was attached at the same position as in acquisition training but no US was delivered. Participants received two startle probes in each of nine CTX^+^ and nine CTX^−^ trials, one startle probe in each of one CTX^+^ and one CTX^−^ trial, and one startle probe during ITI, summing up to overall 58 startle probes (Supplemental Table S3).

Arousal, valence, anxiety, and US expectancy ratings were rated on VAS before and after the acquisition training and after the recall test. For each question, a room image was shown above the VAS anchored with “0” and “100,” and the VAS was partitioned into 10 auxiliary intervals, while responses were given with a continuous slider. For each room, we asked, “How aroused did you feel after experiencing this room? (0 = very calm and 100 = very excited),” “How did you feel after experiencing this room? (0 = very negative and 100 = very positive),” “How anxious did you feel after experiencing this room? (0 = no anxiety at all and 100 = extreme anxiety),” and “When you enter this room do you expect to receive shock/s? (0 = not expected at all and 100 = definitely expected).” CTX–US contingency ratings were collected after acquisition and after recall tasks by asking “How likely were you to receive shock/s in this room today? (0 = never and 100 = always shocked).” Furthermore, after the recall task, participants were asked to recall the contingency in the acquisition task by answering “How likely were you to receive shock/s in this room last week? (0 = never and 100 = always shocked).”

#### Experiments 3 and 4 (configural task)

We adapted a paradigm from Stout et al. (2018, 2019) using the same reinforcement rate as in Stout et al. (2018) and created new room images with https://www.planner5d.com, following the description in Stout et al. (2019). Contextual stimuli were five static room images (Fig. 2B,C), one of which served as CTX^+^ coupled with US, and the other four served as CTX^−^. Different CTX^−^ were used in this paradigm to prevent conditioning to elemental cues. CTX^+^ was the CON_A_^+^ image showing the view of a room with a ceiling, three walls, a floor, and four furniture items. CON_A_^−^ and CON_A_^−^ ER images showed the same room as CON_A_^+^ but either with furniture items rearranged (CON_A_^−^) or with one of the items replaced (CON_A_^−^ ER). The CON_B_^−^ EA and CON_B_^−^ images showed a different room with different furniture. One of the furniture items in CON_B_^−^ EA was the same as in CON_A_^+^. Assignment of room images to CTX^+^/CTX^−^ was the same for every participant.

Participants were instructed to differentiate the room images by room decoration and furniture arrangements. Before acquisition training, participants were shown an overview of the five images on one screen in a random arrangement, followed by a familiarization phase, during which every room image was presented twice in random order without any reinforcement. During acquisition training, participants were presented 88 trials in four blocks. Each block contained six trials of the CTX^+^ condition (i.e., CON_A_^+^) and four trials of each CTX^−^ condition (i.e., CON_A_^−^, CON_A_^−^ ER, CON_B_^−^ EA, and CON_B_^−^). Within each block, the trials were arranged pseudorandomly. The first 10 trials comprised two trials of each CTX in random order, followed by four CTX^+^ trials and two trials of each CTX^−^, again in random order (i.e., 12 trials). In each trial, CTX was presented full screen for 7.5 sec. Five out of six CTX^+^ trials (83%) in each block were reinforced with USs 7.0 sec after trial onset; the first CTX^+^ trial was always reinforced. In reinforced trials, USs coterminated with CTX. A light gray background (RGB [178.5, 178.5, 178.5]) was shown during the ITI with duration determined uniformly at random between 7.5 and 10.5 sec. To help maintain participants’ attention during the task, they were asked to press the “down” arrow key as soon as they had comprehended the room configuration, independent of the type of trial. If they failed to press a key during the CTX, a reminder was shown during the first 2 sec of the ITI. Blocks were separated by a self-paced break. In the recall test, the US electrode was attached at the same position as in the acquisition training while no US was delivered. A startle probe was delivered in every trial (including CTX^−^ trials) at 7.0 sec after trial onset. All other settings in the recall test were the same as in acquisition training (Fig. 2D).

After both acquisition training and recall test, participants rated their arousal, valence, and CTX–US contingency toward each room image on a VAS by answering “How aroused are you when looking at this room? (0 = very calm and 100 = very excited),” “How do you feel when looking at this room? (0 = very unhappy and 100 = very happy),” and “How likely were you to receive an electric shock on your arm when looking at this room today?” After the recall test, participants were further asked to recall the contingency in acquisition training by answering “How likely were you to receive an electric shock on your arm when looking at this room last week?” The corresponding room image was presented on the screen at the top of the question to the participants when the question about “this room” was asked.

### Stimuli and apparatus

#### Experiment presentation

All experiments were conducted in a dark and soundproof chamber. Experiments were coded and displayed in Unity (version 5.5.4f1, Unity Technologies) with a graphics card (NVIDIA GeForce GTX 1080) and presented on a head-mounted display (HMD; 90-Hz refresh rate, display with stereoscopic 3D images at 106.19° horizontally and 95.06° vertically; i.e., 100° diagonally; Oculus Rift HM-A). During VR exploration but not acquisition training or recall test, participants additionally used the hand sensors for free “teleporting” navigation (i.e., movement in VR without body movement). Experiments 3 and 4 (configural paradigm) were coded and displayed with the Cogent 2000 toolbox (v1.32, https://www.vislab.ucl.ac.uk) in Matlab (2019a; The Math Works). Participants were positioned on a chin rest in front of the computer monitor (20 in, aspect ratio 4:3, resolution 1280 × 1024 pixels, refresh rate 60 Hz; Dell P2014h) with a 700-mm eye–monitor distance. Pupil diameter and gaze coordinates in pixels were collected for both eyes with Eyelink 1000 system (SR Research) at a sampling rate of 500 Hz. Calibration and validation of gaze coordinates were completed with the manufacturer's nine-point protocol every time before data recording. The horizontal distance between the eyes and the eye-tracker camera was 470 mm.

STAI and subjective ratings in experiments 1 and 2 were presented in OpenSesame (Mathôt et al. 2012). Subjective ratings in experiments 3 and 4 were presented in Matlab.

#### Unconditioned stimulus

For all experiments, US was an electric shock, delivered to participants’ dominant forearm by a constant current stimulator (Digitimer DS7A) with a pin-cathode/ring-anode configuration. In experiments 1 and 2 (VR paradigm), US was a 0.55-sec train of 25 square pulses with a 0.91% duty cycle. In experiments 3 and 4 (configural paradigm), US was a 0.5-sec train of 250 square pulses with a 10% duty cycle. The intensity of USs was determined individually in a two-phase procedure. (1) In the first staircase phase, we gradually increased electric currents from unperceivable to clearly painful level in steps of 0.3–0.4 mA, until the participants rated a shock as “clearly painful”; that is, the upper limit. (2) In the second random phase, participants received 14 stimuli below the upper limit at random intensities in random order and rated each stimulus on a scale from 0% (no sensation) to 100% (clearly painful). These random intensities corresponded to 40%–100% of the upper limit in steps of 10%; each intensity was rated twice. The final intensity used for the experiment was derived from a linear interpolation of the ratings and corresponded to 85%–90% of the upper limit.

#### Startle probes

Startle probes were white noise sounds of 102-dB loudness with an instantaneous rise time, delivered binaurally with headphones (Sennheiser HD 202). The duration of startle sounds was 50 msec in experiments 1 and 2 to be consistent with the original publication, 20 msec in experiment 3 to minimize the aversive affect of startle probes themselves (Blumenthal et al. 2005), and 40 msec in experiment 4 to increase the sensitivity of SEBRs (Blumenthal and Berg 1986; Putnam and Roth 1990), given that SEBR effect size in experiment 3 was much smaller than expected a priori. One participant in experiment 1 was excluded from the analysis of SEBRs, as well as SCRs toward startle probes, due to technical malfunction of the startle presentation.

#### Data collection

In all experiments, we collected SCRs using a constant voltage coupler/amplifier (Biopac Systems, Inc., EDA100C) from the thenar/hypothenar of the nondominant hand through two disposable pregelled Ag/AgCl snap electrodes (Biopac Systems, Inc., EL507) with an additional layer of 0.5% NaCl gel (Biopac Systems, Inc., GEL 101) (Hygge and Hugdahl 1985). A ground electrode was placed on the nondominant elbow. We collected ECG data with three pregelled Ag/AgCl adhesive snap electrodes (TIGA-MED0 1-7500, Skintact FS-TC1, and Biopac Systems, Inc., EL503) attached to the outsides of wrists and the right ankle, respectively. Lead I configuration yielded clear R spikes for all participants and was recorded (Biopac Systems, Inc., ECG100C). We measured respiration using a single-belt cushion system (Biopac Systems, Inc., RSP100C). In the recall test, electromyogram (EMG) was recorded from the orbicularis oculi muscle of participants’ left eye through two 4-mm shielded Ag/AgCl cup electrodes (Biopac Systems, Inc., EL254S) filled with highly conductive gel (Parker Laboratories, Inc., SignaCreme 17-05). One electrode was placed centrally below the lower eyelid in alignment with the left pupil in forwarding gaze, and the other one was placed laterally below the canthus with a center-to-center distance of ∼1.5–2 cm from the first one (Blumenthal et al. 2005). EMG data were amplified at 2000 Hz with a band-pass filter of 1 and 500 Hz (Biopac Systems, Inc., EMG100C). All data were amplified, digitized (Biopac Systems, Inc., MP160), and recorded with Acknowledge (version 5.0; Biopac Systems, Inc.).

### Data analysis

#### Overview

To avoid overfitting data analysis to a sample of a limited size, we used an exploration conformation approach. For experiments 1 and 3, we explored different psychophysiological indices in their sensitivity to distinguish CTX^+^/CTX^−^. We retained those indices that yielded an effect size of Cohen's *d* > 0.5 and confirmed the effect sizes in experiments 2 and 4. *P*-values are presented without correction for multiple comparisons as a heuristic guide. We did not explore different data analysis pipelines (i.e., inversion parameters), as they were previously optimized in larger samples (Bach et al. 2018a). For heart period responses (HPRs) and respiration amplitude responses (RARs), we did not find any above-threshold effect sizes in any of the experiments; for the sake of space, the details of the analyses are not reported.

As there is no statistical framework to determine sample size to achieve a desired effect size variability, we relied on previous studies in which effect sizes at samples of this size were often relatively similar between samples.

All data preprocessing and model-based analysis of psychophysiological data were conducted in Matlab (R2018b; The Math Works) with the toolbox PsPM (version 4.3.0 for experiments 1 and 2, and version 5.1.1 for experiments 3 and 4; https://bachlab.github.io/pspm). For experiments 3 and 4, where CTX^+^ was partially reinforced by USs, only nonreinforced trials (i.e., CTX^+^ US^−^ and CTX^−^) were included in the analysis to avoid any potential influence of the unconditioned response on our estimates of the conditioned response.

#### Startle eyeblink responses (SEBRs)

EMG preprocessing followed the procedure in Khemka et al. (2017), who optimized filter bands and preprocessing to maximize CTX^+^/CTX^−^ difference in fear-potentiated startle. Data were first filtered by a fourth-order Butterworth filter with cutoff frequencies of 50 and 470 Hz. A 50-Hz notch filter was used to remove mains noise. Data were then rectified and smoothed with a fourth-order Butterworth low-pass filter with a 3-msec time constant. Preprocessed EMG data were visually inspected for quality control (see Supplemental Fig. S8 for an example of quality control). Two participants in experiment 3 had no discernible SEBR in most of the trials and were excluded from SEBR analysis. SEBR amplitude was then estimated using the flexible latency general linear convolution model (GLM) with intervals of 0–150 msec around the startle probe and a canonical response function in PsPM (Khemka et al. 2017). The individual SEBR estimates were normalized in experiments 1 and 2 by dividing through the mean estimate of the participant's ITI trials, and in experiments 3 and 4 by dividing through the average of all CTX^−^ trials.

#### Skin conductance responses (SCRs)

SCR data were visually inspected. One participant in experiment 1 had no discernible SCR toward the US and was excluded from all SCR analyses.

In experiments 1 and 2, SCR data were filtered unidirectionally with cutoff frequencies of 0.05–5 Hz and down-sampled to 10 Hz. We derived the following indices: tonic skin conductance level (SCL) in each trial during the acquisition training (across the entire time spent in a room), phasic SCRs elicited at trial onset (i.e., room or corridor entry) in the acquisition training and the recall test, and phasic SCRs elicited by startle probes during the recall test. SCRs elicited at trial onset and by startle probes were estimated on a trial-by-trial basis using the GLM approach implemented in PsPM with a standard canonical skin conductance response function (Bach et al. 2010b). Resulting SCL and SCR estimates were normalized by dividing through the average estimates from that participants’ ITI.

In experiments 3 and 4, SCR data were filtered with a first-order bidirectional band-pass Butterworth filter (cutoff frequencies 0.0159 Hz–5 Hz) and down-sampled to 10 Hz, similar to previous work (Staib et al. 2015). We then estimated conditioned and unconditioned responses using the nonlinear model implemented in PsPM (dynamic causal modeling [DCM]) (Bach et al. 2010a; Staib et al. 2015). We modeled a response to CTX onset (fixed onset and dispersion of 0.3 sec) and a response during the CTX (between CTX onset and 1.5 sec before US time point; fixed dispersion of 0.3 sec). The resulting estimates were normalized by dividing through the average of all CTX^−^ trials from the corresponding participant.

#### Pupil dilation

Pupil size in diameter was converted from arbitrary Eyelink unit system to millimeters in PsPM using the transform provided in PsPM (Korn and Bach 2016). Pupil size data from both eyes were then preprocessed and combined following the procedure by Kret and Sjak-Shie (2019) as implemented in PsPM. Because of the size of the CTX images, which exceeded foveal vision and thus required participants to move their eyes, we corrected apparent pupil size for gaze deviation (Hayes and Petrov 2016). Data points with gaze coordinates exceeding ±9.2654° visual angle (corresponding to the entire vertical extension of the screen and the horizontal area of interest in the room images) were excluded. We estimated trial-by-trial pupil size in the standard GLM approach with fear conditioning pupil response function as implemented in PsPM (Korn and Bach 2016; Korn et al. 2017). To account for different luminance between the five CTX images, we created time series of the predicted illuminance response corresponding to the visual input and used these as a nuisance regressor in the GLM (Korn and Bach 2016). Missing data during a trial can lead to unidentifiable amplitude; hence, trials with unreasonable pupil dilation estimates (i.e., estimates exceeding ±6 mm) (Spector 1990) after GLM were excluded. We ensured that each participant had >50% usable trials. Across participants, >99% of trials were usable and included.

### Statistical analysis

#### Outlier rejection

For condition-wise data, participants with data points outside three standard deviations of the corresponding condition group mean were excluded from the analysis and figures. This excluded one participant from analysis of SCRs to trial onset and one from SCR analysis during CTX in each of experiments 3 and 4. For trial-wise data, data points outside three standard deviations around the group mean for this trial and condition were excluded. Participants with >50% of trials excluded were removed from further analysis and figures. This excluded one participant in experiment 2 from SEBR analysis.

#### Statistical modeling

##### General

SCR and SEBR indices were normalized within subject as described above. No further data transformation was conducted on these or other indices before statistical analysis. For all trial-wise indices (SCRs, pupil dilation, and SEBRs), our primary analysis was a linear mixed-effect (LME) model [function lmerTest::lmer()] in R (version 3.6.2 for experiments 1 and 2 and version 4.1.0 for experiments 3 and 4; https://www.r-project.org), with formulae as specified below. As there is no established effect size estimator for LMEs, we used the effect size from post-hoc *t*-tests of condition averages to determine inclusion in further analysis. For acquisition training, we averaged over all trials from each condition. For the recall test, CTX^+^/CTX^−^ differences might diminish during the test that was done without reinforcement (such that extinction might take place). Hence, we averaged over a different number of trials to determine an optimal memory retention index.

For condition-wise indices (HPRs, RARs, and subjective ratings), our primary analysis was a one-way or two-way repeated-measures ANOVA [rmANOVA, function aov()] with factors as specified below and Greenhouse–Gerisser correction for lack of sphericity whenever needed [function rstatix::anova_test()]. Descriptive results are reported as group mean ± standard error unless specified.

##### Experiments 1 and 2

For SCRs to trial onset, we used the LME formula data ∼ conditions × trial_index + (1/subject), where condition is a factor with three levels (CTX^+^/CTX^−^/ITI) and trial_index is a linear (numerical) index of trial. For responses to startle probes, we used the LME formula data ∼ conditions × index × probe_within_trial + (1/subject), where index is the probe number over the whole experiment in the recall test (i.e., 1–58) and probe_within_trial is the probe number within a trial (1 or 2). Linear trial/probe indices were added to account for the main and interaction effects of time; for example, the dynamic learning process and potential response habituation. For HPRs and RARs, we used a one-way ANOVA with the factor condition CTX^+^/CTX^−^/ITI. For SCL, CTX^+^ and CTX^−^ were compared in paired *t*-tests. To investigate methods of creating a memory retention index, we averaged the first *N* trials of each condition and compared CTX^+^ with CTX^−^ in paired *t*-tests.

##### Experiments 3 and 4

We used the LME formula data ∼ conditions × trial_index + (1/subject), where condition is a factor with five levels (for the individual room images) and trial_index is a linear (numerical) index of trial. Linear trial/probe indices were added to account for the main and interaction effects of time; for example, the dynamic learning process and potential response habituation. To investigate methods of creating a memory retention index, we again averaged the first *N* trials of each condition. Since the positions of CTX^+^ and CTX^−^ trials were unbalanced in the second part of each block, such that habituation might have a different effect for each condition, we sorted trials in each block into four subsets. For each block, the first and the second subset contained the first and the second trial of each condition, respectively, with fully randomized positions across participants. The third subset contained the average of the third and the fourth CTX^+^ trials and the third trial of each CTX^−^, and the last subset contained the average of the last two CTX^+^ and the last CTX^−^ trials. In total, four subsets per block were balanced in timing. We then averaged the first *N* subsets of each condition and compared CTX^+^ with averaged CTX^−^ in 16 post-hoc paired *t*-tests.

##### Subjective ratings

In all experiments, subjective ratings were analyzed in rmANOVA {formula: data ∼ rating_time_point × condition + error[subject/(rating_time_point × condition)]}. In experiment 1, preacquisition ratings from one participant and the preacquisition arousal rating from another participant were missing.

##### Effect sizes

Effect sizes of rmANOVA were calculated as partial η^2^ in R [function rstatix::anova_summary()], and those of paired *t*-tests were quantified as Hedge's *g*. Hedge's *g* was calculated manually in Matlab (R2018b) with the following formula (Lakens 2013; Hedges and Olkin 2014):

$$g=J(n-1)*\frac{\bar{Xdiff}}{SDdiff},\mathrm{where}\;J(a)=\frac{\Gamma \left(\frac{a}{2}\right)}{\sqrt{\frac{a}{2}}\;\Gamma \left(\frac{a-1}{2}\right)}.$$

### Data Deposition

All data sets are available in anonymized form at https://www.zenodo.org (Table 1). Supplemental material, experimental codes, and analysis scripts are available from OSF (https://doi.org/10.17605/OSF.IO/4MXAE).

### Competing interest statement

The authors declare no competing interests.

## Supplementary Material

Supplement 1
