# Supplementary material for: Measuring human context fear conditioning and retention after consolidation
Source: Learn Mem. 2023 Jul;30(7):139–50. doi: 10.1101/lm.053781.123 (PMC10519410; doi:10.1101/lm.053781.123)
Supplement: Supplement 1 [file Supplemental_Material.docx]

1. Experiment 1


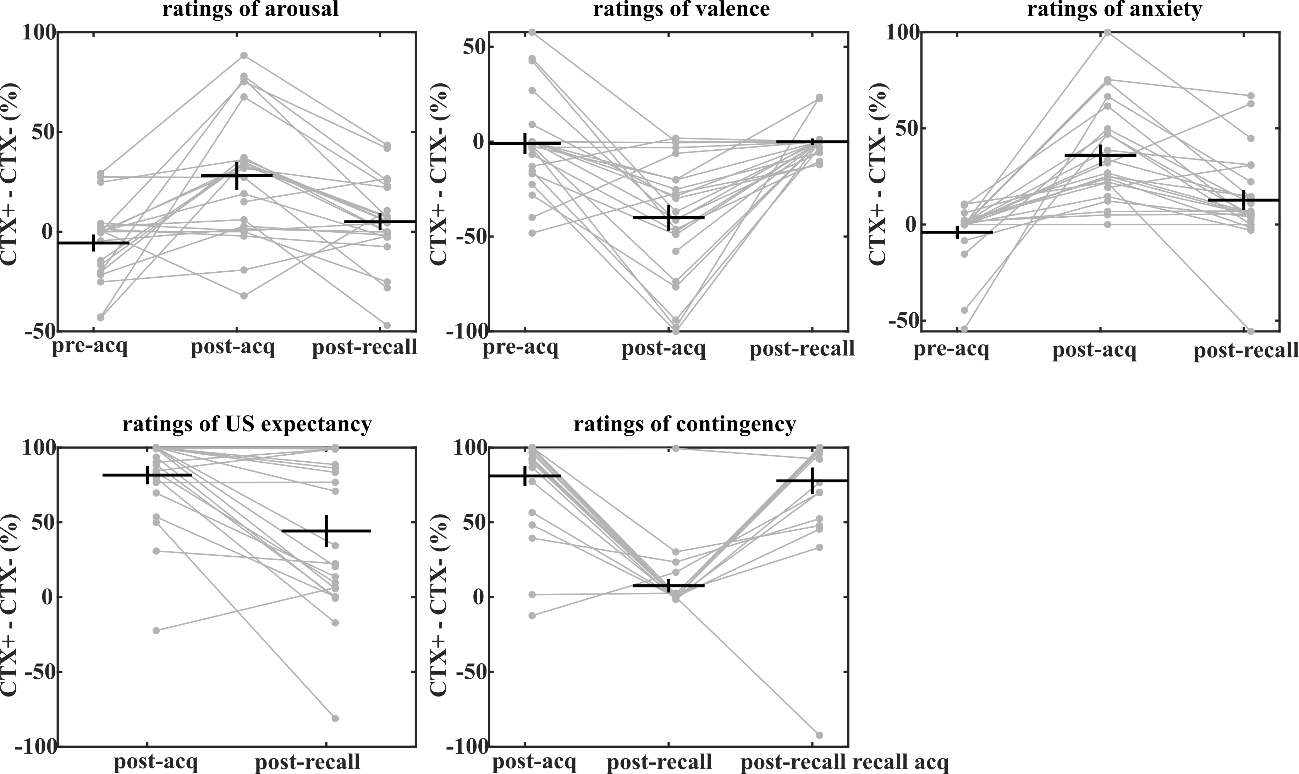


1. Experiment 2


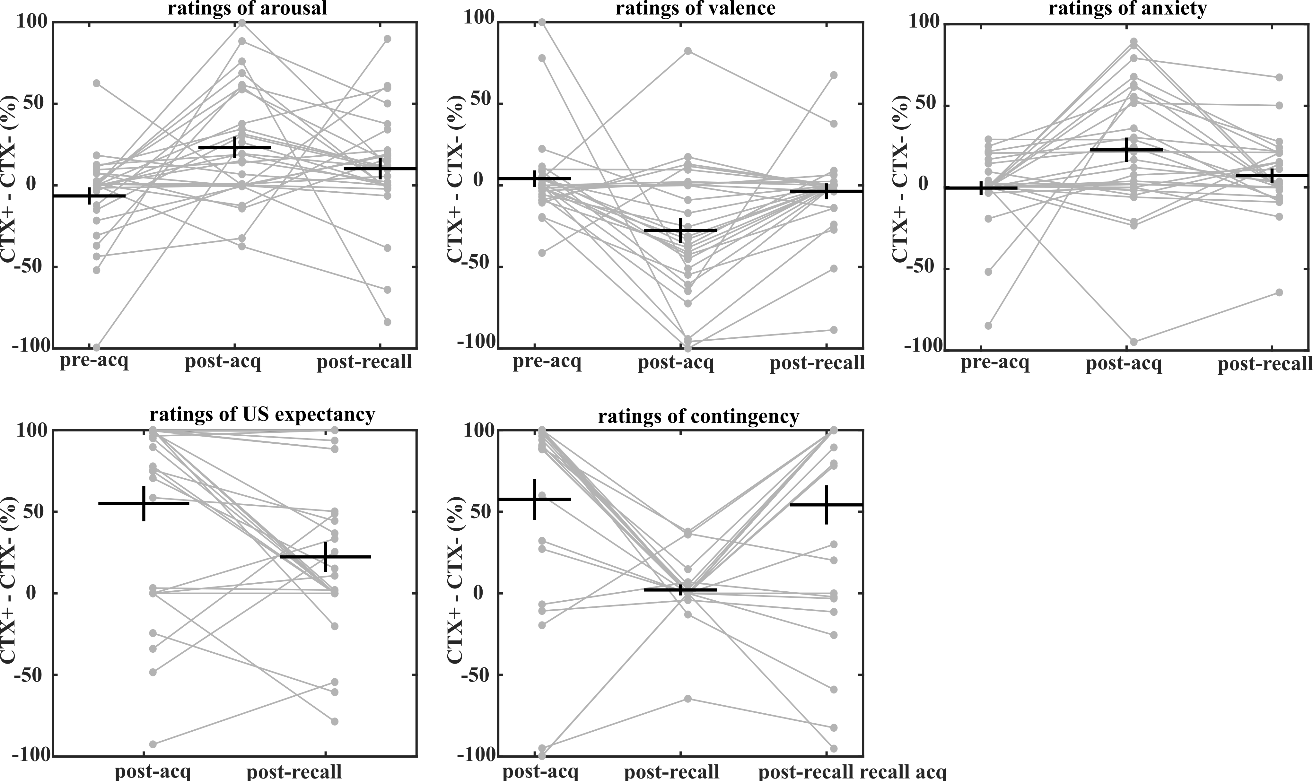


Figure S1. Subjective ratings in experiment 1 (A) and 2 (B). Individual data is plotted in grey and group data is plotted in black as group mean ± SEM.

1. Experiment 3 acquisition training


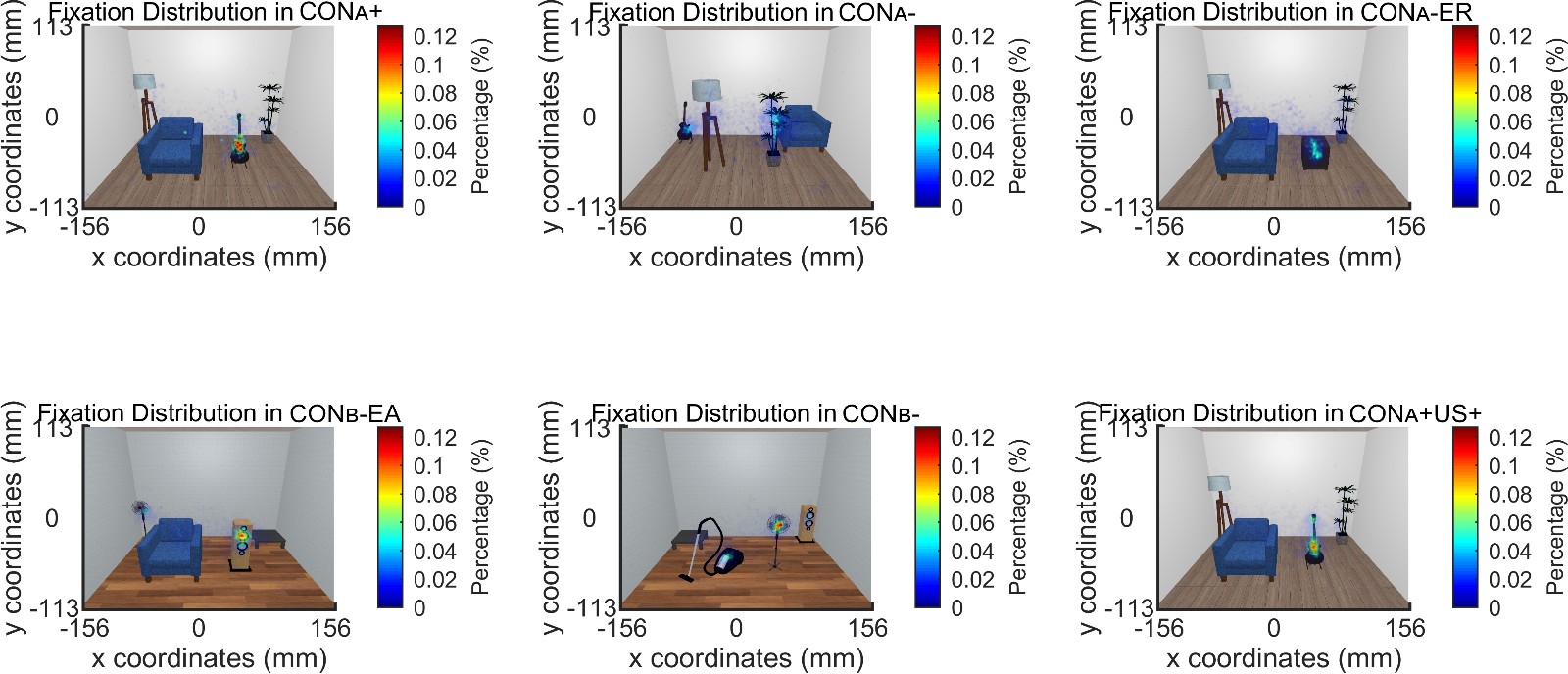


1. Experiment 3 recall test


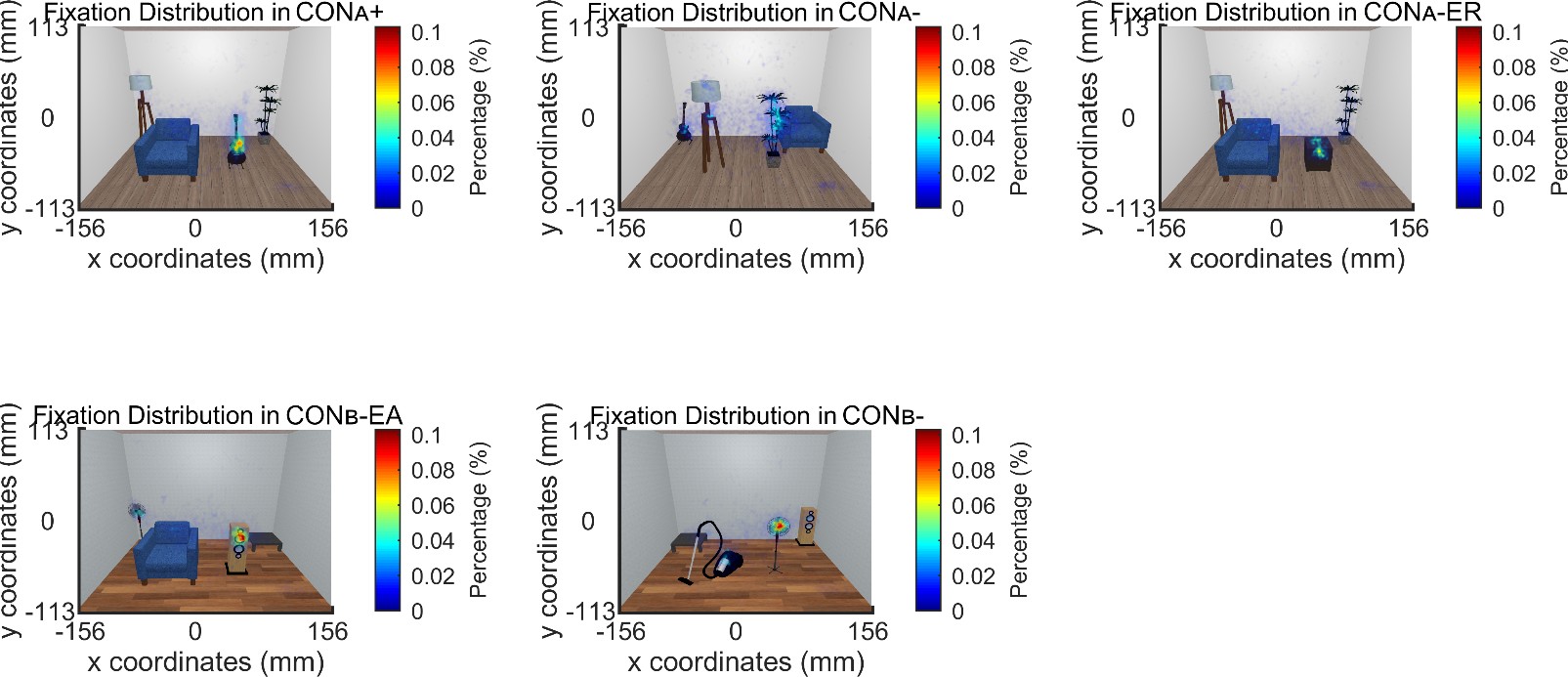


Figure S2. Fixation distribution in experiment 3. (A) During memory acquisition. (B) During memory recall. Gaze coordinates are plotted in a scale of the presentation screen.

1. Experiment 4 acquisition training


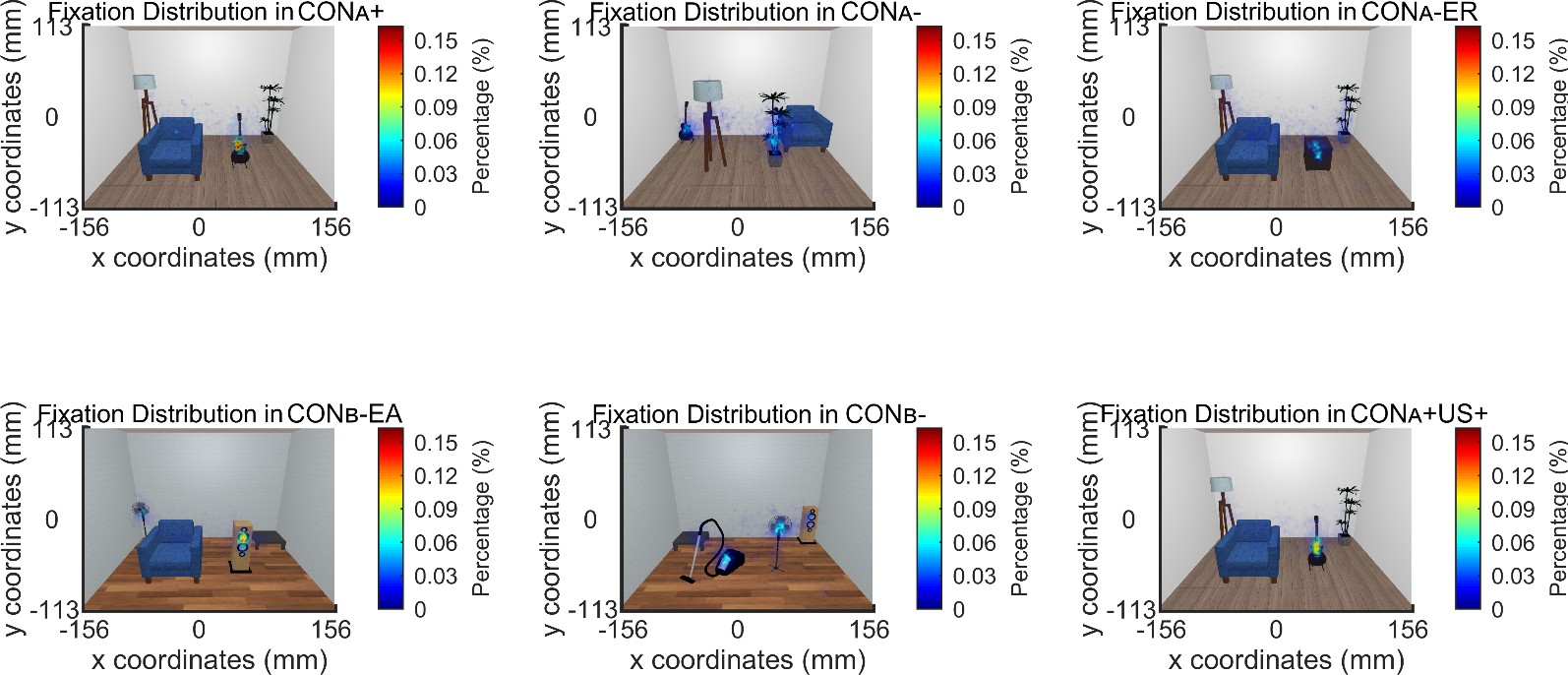


1. Experiment 4 recall test


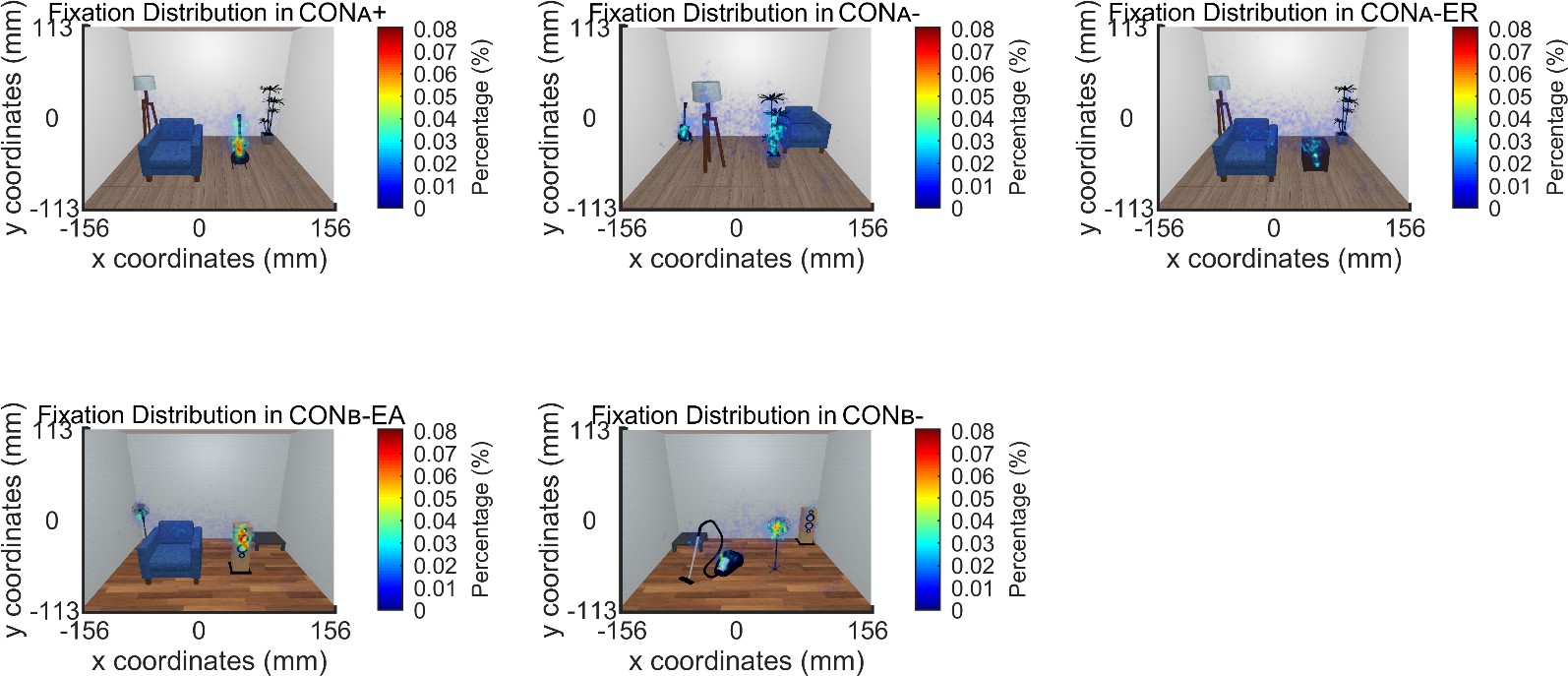


Figure S3. Fixation distribution in experiment 4. (A) During memory acquisition. (B) During memory recall. Gaze coordinates are plotted in a scale of the presentation screen.


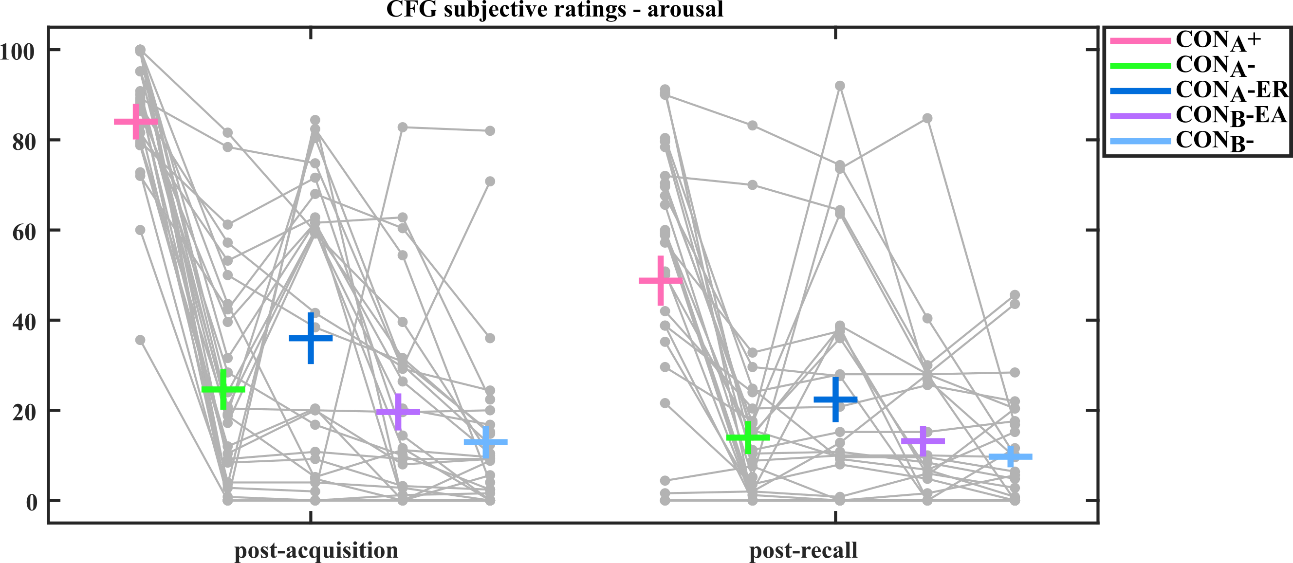


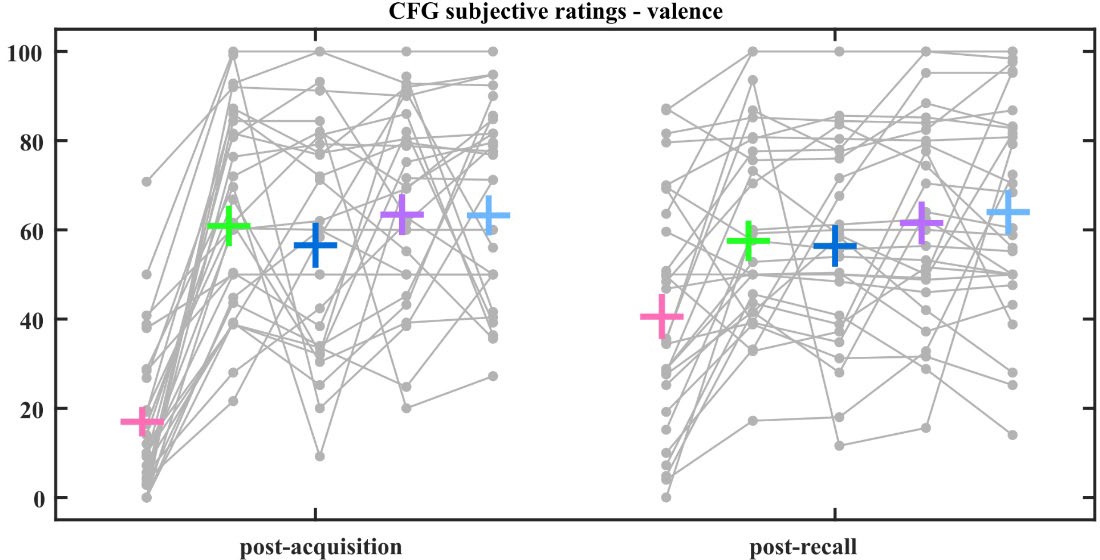


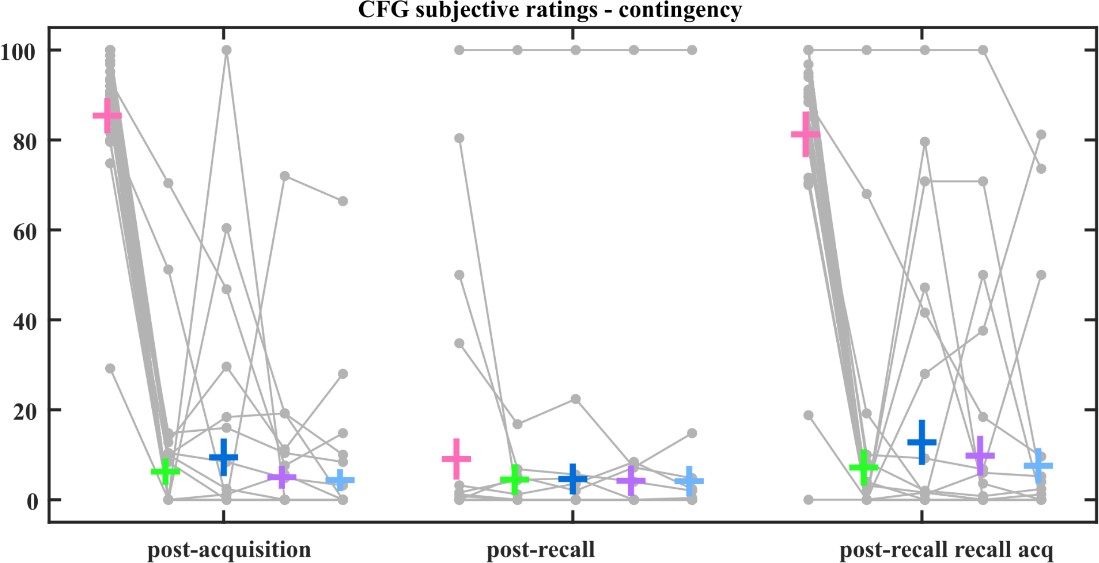


Figure S4. Subjective ratings in experiment 3. Individual data is plotted in grey and group data is plotted in corresponding colors as group mean ± SEM.


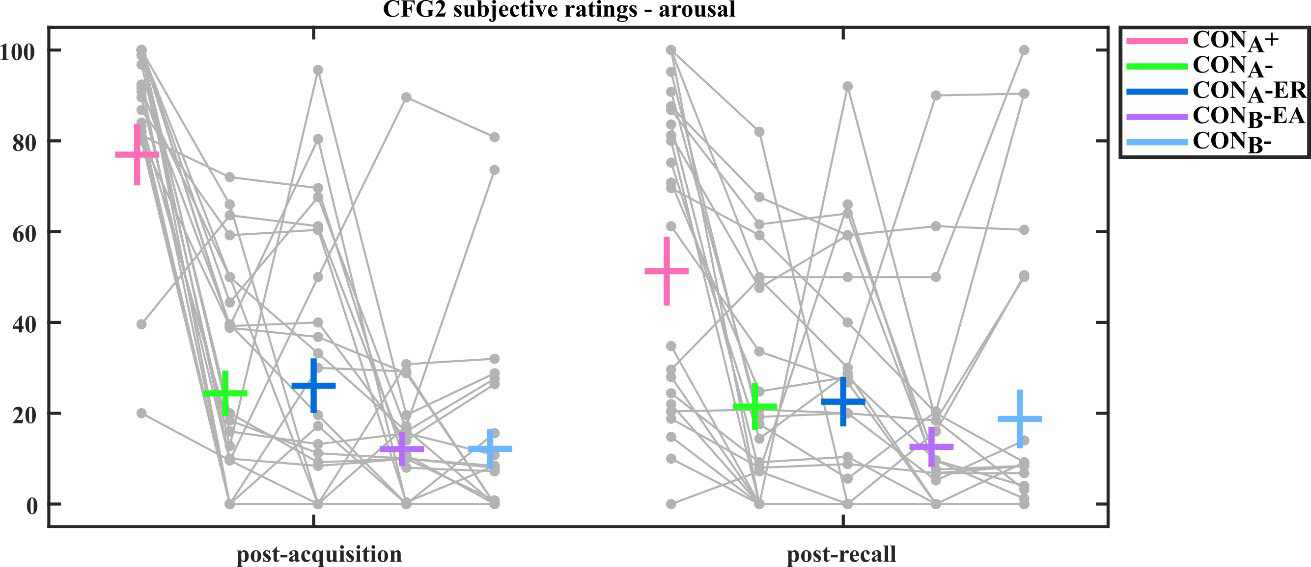


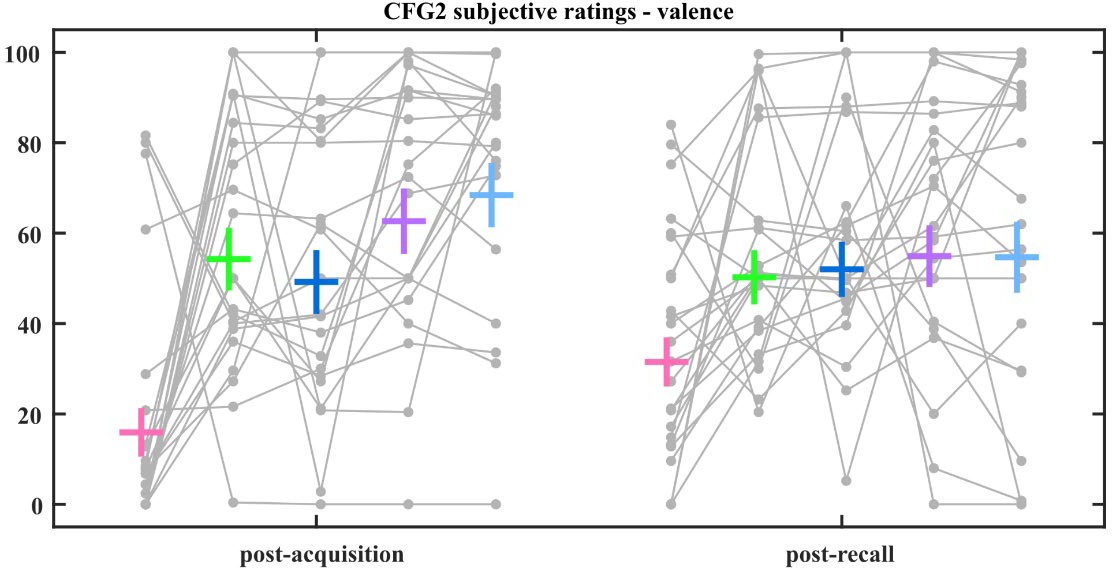


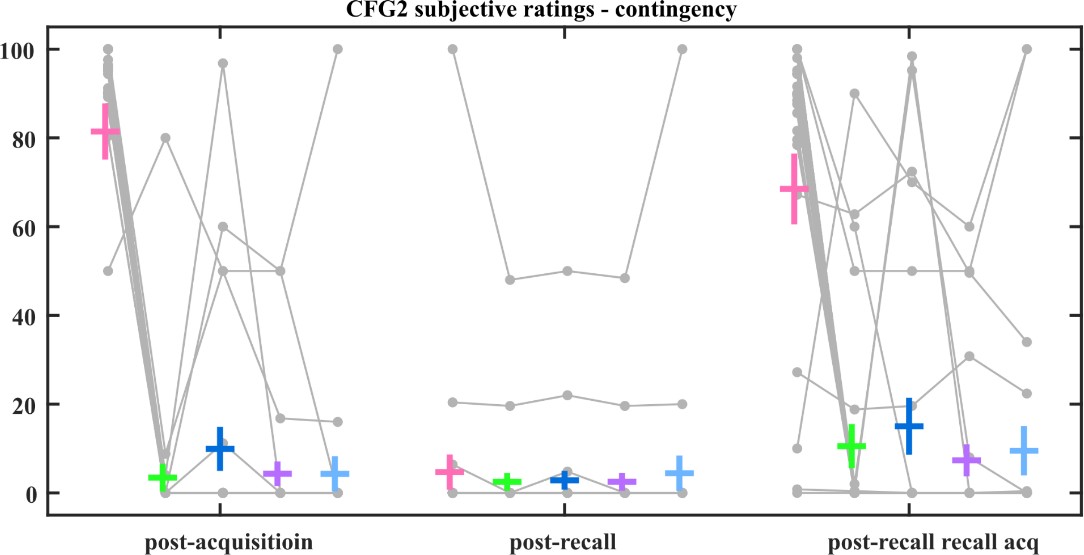


Figure S5. Subjective ratings in experiment 4. Individual data is plotted in grey and group data is plotted in corresponding colors as group mean ± SEM.

1. Experiment 3


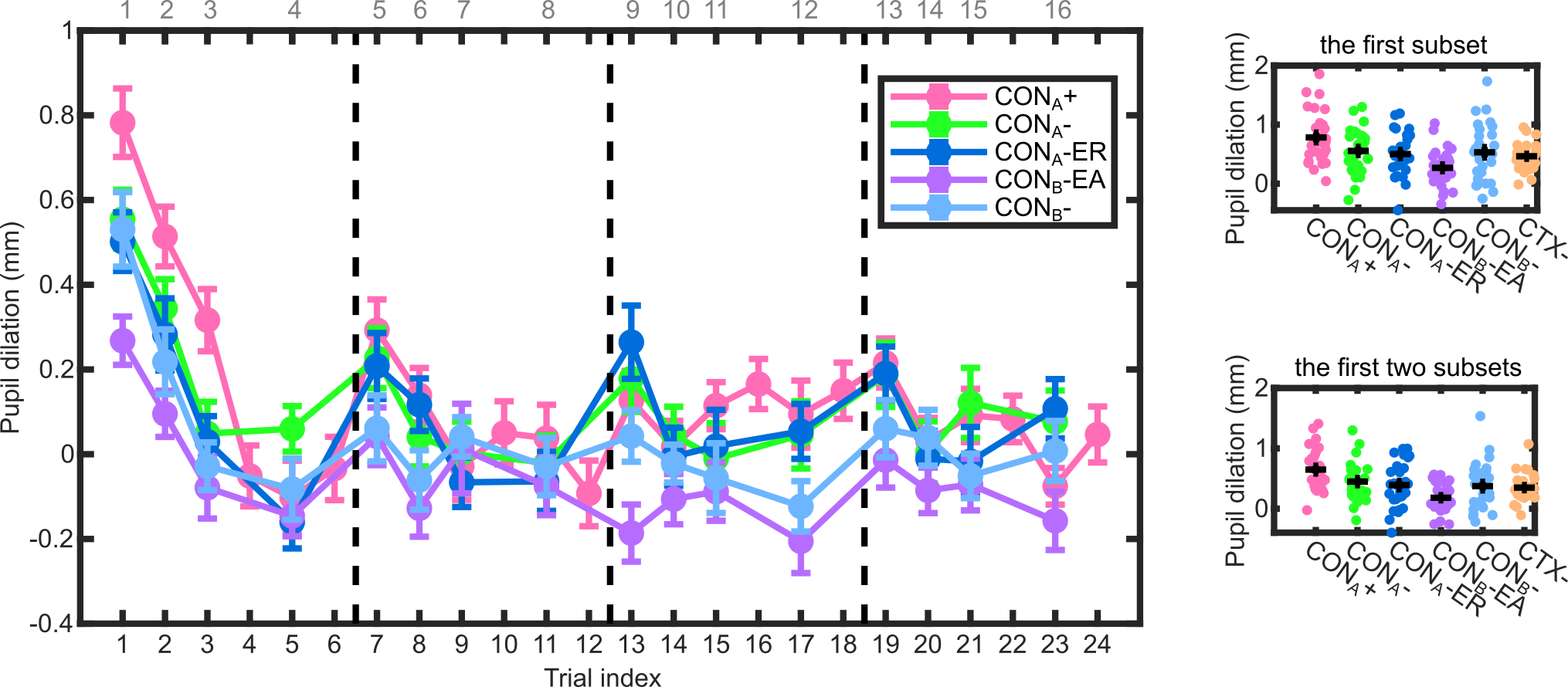


1. Experiment 4


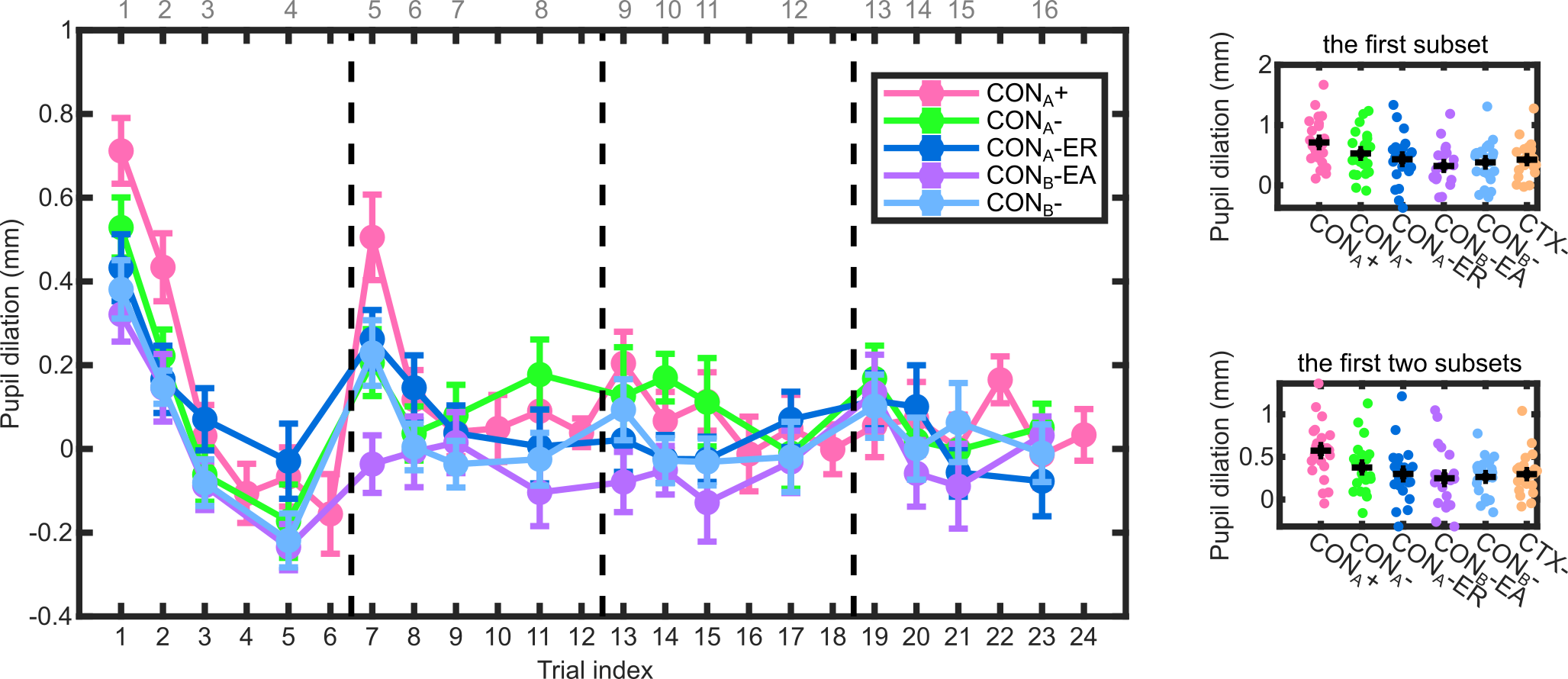


Figure S6. Pupil dilation during memory recall in experiment 3 (A) and 4 (B). Trial-wise pupil dilation is plotted on left with dashed black lines separating blocks. X axis on the top represents trial index of CTX- conditions. The average over the first one and the first two subsets are plotted on right. LME analysis suggests a trial effect on pupil dilation, and larger pupil dilation in CONA+ than in CONB- and in CONB-EA in both experiments; explorative paired t-test on average of the first N subsets supports LME results. Paired t-test also shows notable difference between CONA+ and CONA- and between CONA+ and CONA-ER in the first and the second subsets. Group data is plotted in corresponding colors as group mean ± SEM. Individual data is plotted in colored dots. “CTX-” represents data averaged over all four CTX- conditions.

- 1. Experiment 3


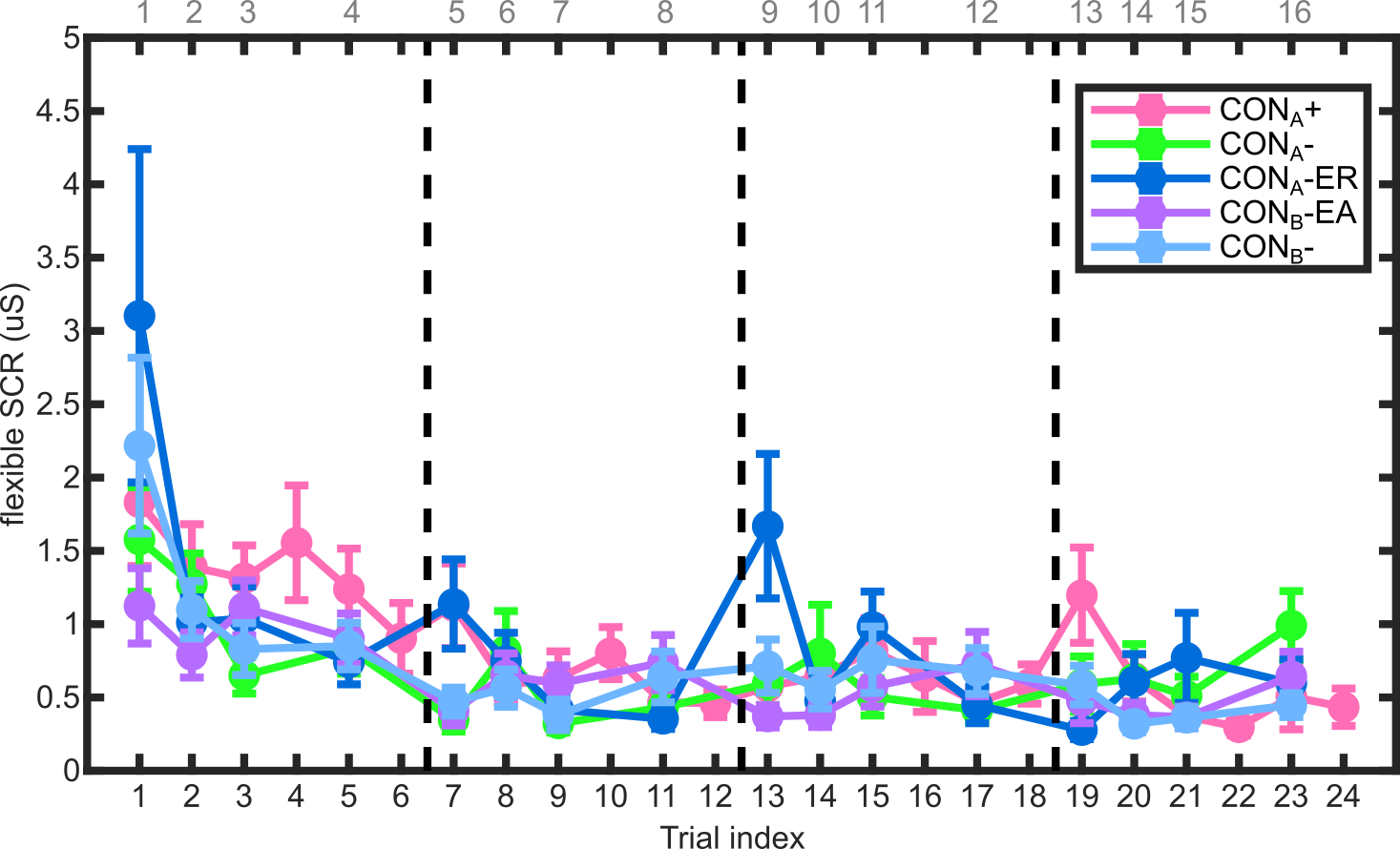


- 1. Experiment 4


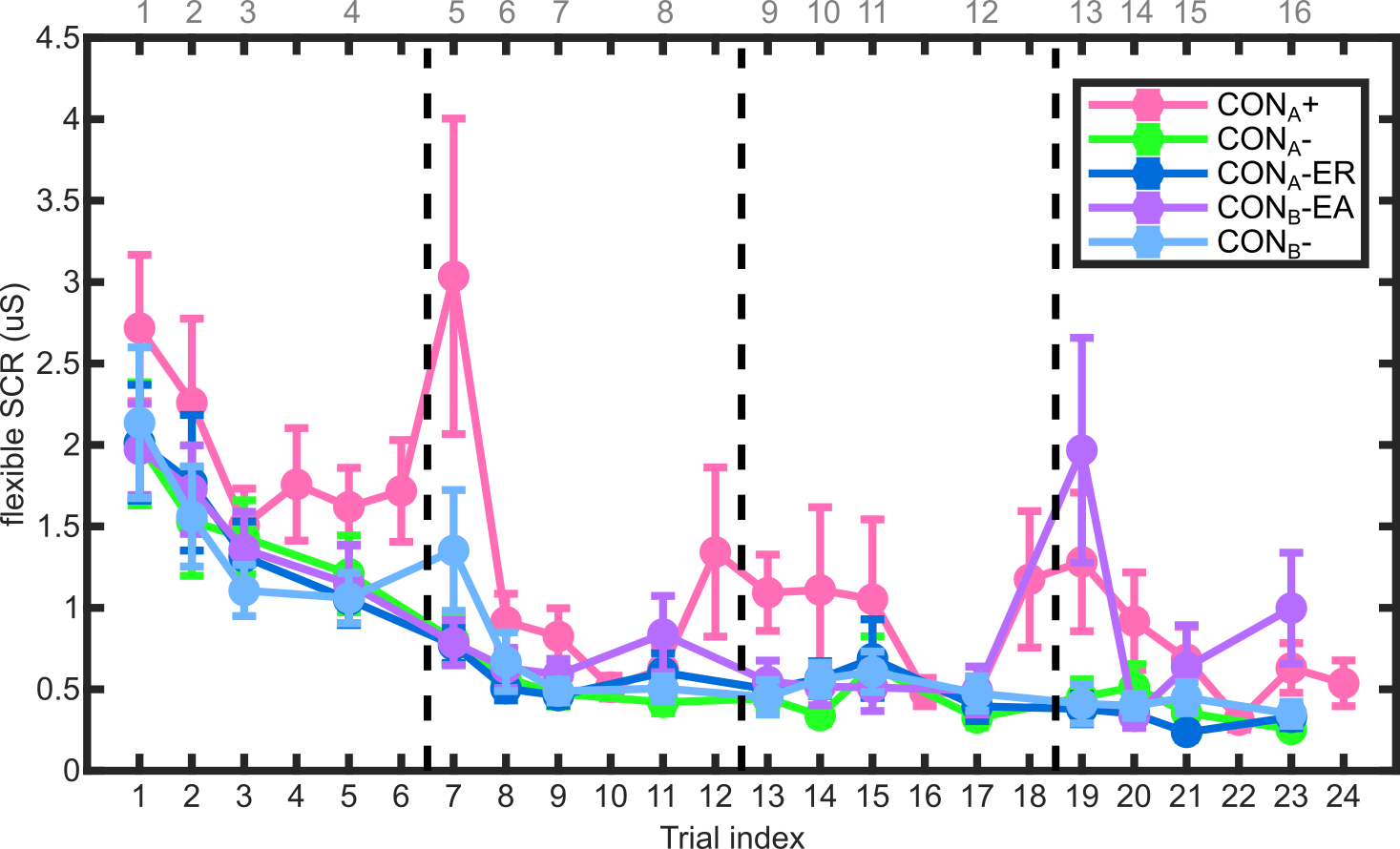


Figure S7. Normalised SCR estimates flexible during CTX presentation during memory recall in experiment 3 (A) and 4 (B). Trial-wise SCR is plotted with dashed black lines separating blocks. X axis on the top represents trial index of CTX- conditions. Although LME and paired t-test results support each other in each experiment, they does not show consistency between experiments. Data is plotted in corresponding colors as group mean ± SEM.

A.


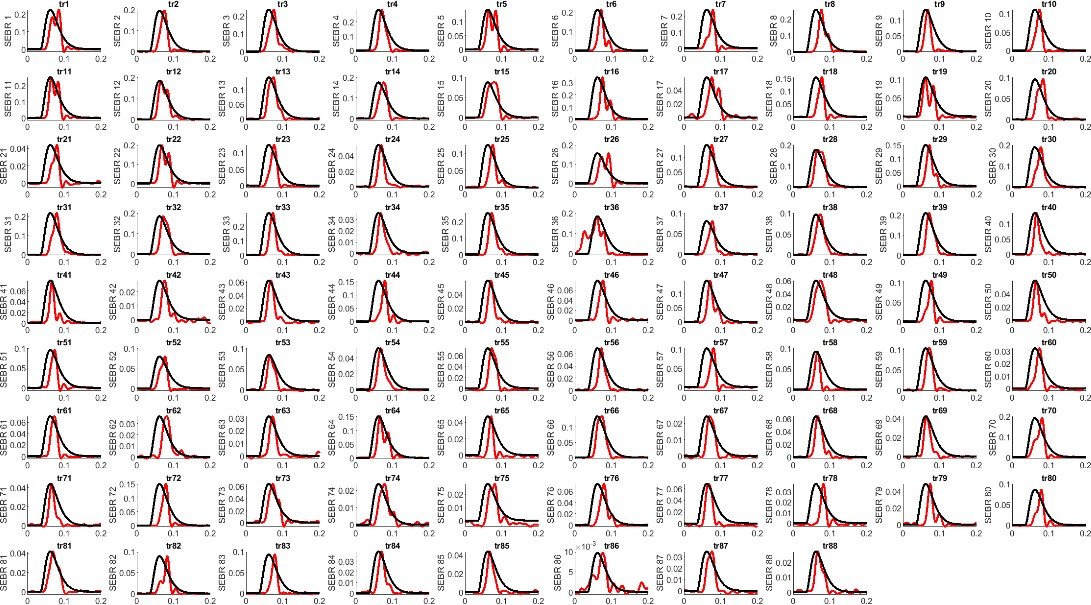


B.


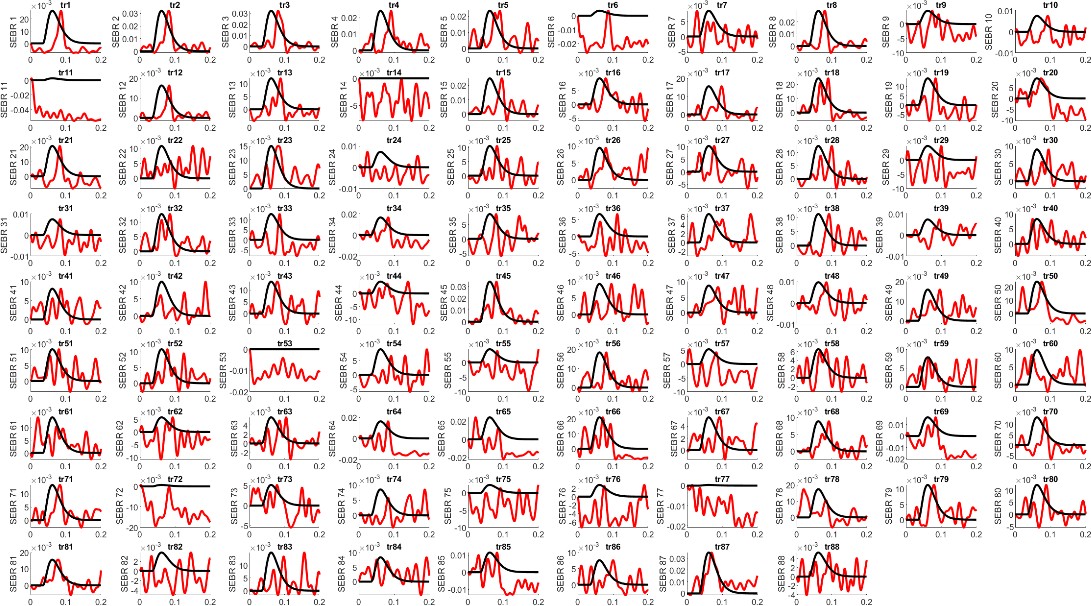


Figure S8. Examples of visual inspectation for SEBR quality control. (A) An example of a participant with discernible SEBR in most trials. (B) An example of a participant without discernible SEBR in most trials. Red curves were raw EMG data within a 0- 200 ms interval after startle probe delivery; black curves were the scaled standard canonical response function implemented in PsPM. Two participants in experiment 3 had no discernible SEBR in most of the trials and were excluded from SEBR analysis.

| Table S1 CTX paradigm acquisition design 1 | | | | | | |
| --- | --- | --- | --- | --- | --- | --- |
| **case** | **rooms** | **n. of probes** | **n. of shocks** | **CTX** | **duration (/s)** | **block_number** |
| 1 | Hallway | 0 | 0 | ITI | 13 | 1 |
| 2 | Vase | 0 | 2 | + | 32 | 1 |
| 3 | Hallway | 0 | 0 | ITI | 13 | 1 |
| 4 | Chairs | 0 | 0 | - | 32 | 1 |
| 5 | Hallway | 0 | 0 | ITI | 13 | 1 |
| 6 | Vase | 0 | 1 | + | 32 | 1 |
| 7 | Hallway | 0 | 0 | ITI | 13 | 1 |
| 8 | Chairs | 0 | 0 | - | 32 | 1 |
| 9 | Hallway | 0 | 0 | ITI | 13 | 1 |
| 10 | Chairs | 0 | 0 | - | 32 | 1 |
| 11 | Hallway | 0 | 0 | ITI | 13 | 1 |
| 12 | Vase | 0 | 2 | + | 32 | 1 |
| 13 | Hallway | 0 | 0 | ITI | 13 | 1 |
| 14 | Chairs | 0 | 0 | - | 32 | 1 |
| 15 | Hallway | 0 | 0 | ITI | 13 | 1 |
| 16 | Vase | 0 | 1 | + | 32 | 1 |
| 17 | Hallway | 0 | 0 | ITI | 13 | 1 |
| 18 | Vase | 0 | 2 | + | 32 | 1 |
| 19 | Hallway | 0 | 0 | ITI | 13 | 1 |
| 20 | Chairs | 0 | 0 | - | 32 | 1 |
| 21 | Hallway | 0 | 0 | ITI | 13 | 1 |
| 22 | Chairs | 0 | 0 | - | 32 | 1 |
| 23 | Hallway | 0 | 0 | ITI | 13 | 1 |
| 24 | Vase | 0 | 2 | + | 32 | 1 |
| 25 | Hallway | 0 | 0 | ITI | 13 | 1 |
| 26 | Chairs | 0 | 0 | - | 32 | 1 |
| 27 | Hallway | 0 | 0 | ITI | 13 | 1 |
| 28 | Vase | 0 | 2 | + | 32 | 1 |
| 29 | Hallway | 0 | 0 | ITI | 13 | 1 |
| 30 | Chairs | 0 | 0 | - | 32 | 1 |
| 31 | Hallway | 0 | 0 | ITI | 13 | 1 |
| 32 | Vase | 0 | 1 | + | 32 | 1 |
| 33 | Hallway | 0 | 0 | ITI | 13 | 1 |
| 34 | Vase | 0 | 2 | + | 32 | 1 |
| 35 | Hallway | 0 | 0 | ITI | 13 | 1 |
| 36 | Chairs | 0 | 0 | - | 32 | 1 |
| 37 | Hallway | 0 | 0 | ITI | 13 | 1 |
| 38 | Vase | 0 | 1 | + | 32 | 1 |
| 39 | Hallway | 0 | 0 | ITI | 13 | 1 |
| 40 | Chairs | 0 | 0 | - | 32 | 1 |
| 41 | End | 0 | 0 | ITI |  | 1 |

| Table S2 CTX paradigm acquisition design 2 | | | | | | |
| --- | --- | --- | --- | --- | --- | --- |
| **case** | **rooms** | **n. of probes** | **n. of shocks** | **CTX** | **duration (/s)** | **block_number** |
| 1 | Hallway | 0 | 0 | ITI | 13 | 1 |
| 2 | Vase | 0 | 0 | - | 32 | 1 |
| 3 | Hallway | 0 | 0 | ITI | 13 | 1 |
| 4 | Chairs | 0 | 2 | + | 32 | 1 |
| 5 | Hallway | 0 | 0 | ITI | 13 | 1 |
| 6 | Vase | 0 | 0 | - | 32 | 1 |
| 7 | Hallway | 0 | 0 | ITI | 13 | 1 |
| 8 | Chairs | 0 | 1 | + | 32 | 1 |
| 9 | Hallway | 0 | 0 | ITI | 13 | 1 |
| 10 | Chairs | 0 | 2 | + | 32 | 1 |
| 11 | Hallway | 0 | 0 | ITI | 13 | 1 |
| 12 | Vase | 0 | 0 | - | 32 | 1 |
| 13 | Hallway | 0 | 0 | ITI | 13 | 1 |
| 14 | Chairs | 0 | 1 | + | 32 | 1 |
| 15 | Hallway | 0 | 0 | ITI | 13 | 1 |
| 16 | Vase | 0 | 0 | - | 32 | 1 |
| 17 | Hallway | 0 | 0 | ITI | 13 | 1 |
| 18 | Vase | 0 | 0 | - | 32 | 1 |
| 19 | Hallway | 0 | 0 | ITI | 13 | 1 |
| 20 | Chairs | 0 | 2 | + | 32 | 1 |
| 21 | Hallway | 0 | 0 | ITI | 13 | 1 |
| 22 | Chairs | 0 | 2 | + | 32 | 1 |
| 23 | Hallway | 0 | 0 | ITI | 13 | 1 |
| 24 | Vase | 0 | 0 | - | 32 | 1 |
| 25 | Hallway | 0 | 0 | ITI | 13 | 1 |
| 26 | Chairs | 0 | 2 | + | 32 | 1 |
| 27 | Hallway | 0 | 0 | ITI | 13 | 1 |
| 28 | Vase | 0 | 0 | - | 32 | 1 |
| 29 | Hallway | 0 | 0 | ITI | 13 | 1 |
| 30 | Chairs | 0 | 1 | + | 32 | 1 |
| 31 | Hallway | 0 | 0 | ITI | 13 | 1 |
| 32 | Vase | 0 | 0 | - | 32 | 1 |
| 33 | Hallway | 0 | 0 | ITI | 13 | 1 |
| 34 | Vase | 0 | 0 | - | 32 | 1 |
| 35 | Hallway | 0 | 0 | ITI | 13 | 1 |
| 36 | Chairs | 0 | 2 | + | 32 | 1 |
| 37 | Hallway | 0 | 0 | ITI | 13 | 1 |
| 38 | Vase | 0 | 0 | - | 32 | 1 |
| 39 | Hallway | 0 | 0 | ITI | 13 | 1 |
| 40 | Chairs | 0 | 1 | + | 32 | 1 |
| 41 | End | 0 | 0 | ITI |  | 1 |

| Table S3 CTX paradigm extinction design | | | | | | |
| --- | --- | --- | --- | --- | --- | --- |
| **case** | **rooms** | **n. of probes** | **n. of shocks** | **CTX** | **duration (/s)** | **block_number** |
| 1 | Hallway | 1 | 0 | ITI | 13 | 1 |
| 2 | Vase | 2 | 0 | - | 32 | 1 |
| 3 | Hallway | 1 | 0 | ITI | 13 | 1 |
| 4 | Chairs | 2 | 0 | - | 32 | 1 |
| 5 | Hallway | 1 | 0 | ITI | 13 | 1 |
| 6 | Vase | 2 | 0 | - | 32 | 1 |
| 7 | Hallway | 1 | 0 | ITI | 13 | 1 |
| 8 | Chairs | 2 | 0 | - | 32 | 1 |
| 9 | Hallway | 1 | 0 | ITI | 13 | 1 |
| 10 | Chairs | 2 | 0 | - | 32 | 1 |
| 11 | Hallway | 1 | 0 | ITI | 13 | 1 |
| 12 | Vase | 2 | 0 | - | 32 | 1 |
| 13 | Hallway | 1 | 0 | ITI | 13 | 1 |
| 14 | Chairs | 2 | 0 | - | 32 | 1 |
| 15 | Hallway | 1 | 0 | ITI | 13 | 1 |
| 16 | Vase | 2 | 0 | - | 32 | 1 |
| 17 | Hallway | 1 | 0 | ITI | 13 | 1 |
| 18 | Vase | 2 | 0 | - | 32 | 1 |
| 19 | Hallway | 1 | 0 | ITI | 13 | 1 |
| 20 | Chairs | 2 | 0 | - | 32 | 1 |
| 21 | Hallway | 1 | 0 | ITI | 13 | 1 |
| 22 | Chairs | 2 | 0 | - | 32 | 1 |
| 23 | Hallway | 1 | 0 | ITI | 13 | 1 |
| 24 | Vase | 2 | 0 | - | 32 | 1 |
| 25 | Hallway | 1 | 0 | ITI | 13 | 1 |
| 26 | Chairs | 1 | 0 | - | 32 | 1 |
| 27 | Hallway | 1 | 0 | ITI | 13 | 1 |
| 28 | Vase | 2 | 0 | - | 32 | 1 |
| 29 | Hallway | 1 | 0 | ITI | 13 | 1 |
| 30 | Chairs | 2 | 0 | - | 32 | 1 |
| 31 | Hallway | 1 | 0 | ITI | 13 | 1 |
| 32 | Vase | 2 | 0 | - | 32 | 1 |
| 33 | Hallway | 1 | 0 | ITI | 13 | 1 |
| 34 | Vase | 1 | 0 | - | 32 | 1 |
| 35 | Hallway | 1 | 0 | ITI | 13 | 1 |
| 36 | Chairs | 2 | 0 | - | 32 | 1 |
| 37 | Hallway | 1 | 0 | ITI | 13 | 1 |
| 38 | Vase | 2 | 0 | - | 32 | 1 |
| 39 | Hallway | 1 | 0 | ITI | 13 | 1 |
| 40 | Chairs | 2 | 0 | - | 32 | 1 |
| 41 | End | 0 | 0 | ITI |  | 1 |

| Table S4 |  |  |  |  |
| --- | --- | --- | --- | --- |
| Experiment 1 and 2 SSQ | | | | |
| **time** | **Nausea** | **Oculomotor** | **Disorientation** | **SSQ total** |
| Exp 1, acq | 40.6 ± 38.4 ^**^ | 30.3 ± 26.8 ^**^ | 44.8 ± 44.4 ^**^ | 42.9 ± 36.9 ^**^ |
| Exp 1, recall | 16.2 ± 17.8 ^*^ | 19.1 ± 15.8 ^*^ | 16.3 ± 24.7 ^*^ | 20.2 ± 17.4 ^*^ |
| Exp 2, acq | 40.9 ± 39.8 ^**^ | 31.9 ± 23.1 ^**^ | 55.7 ± 47.5 ^**^ | 46.8 ± 36.5 ^**^ |
| Exp 2, recall | 37.8 ± 30.6 ^**^ | 34.4 ± 22.2 ^**^ | 44.2 ± 40.3 ^**^ | 43.7 ± 31.5 ^**^ |

| a. data shown in mean +/- sd |
| --- |
| b. * higher than calibration sample, p < .05; ** higher than calibration sample, p < .01 |

| Table S5 | Experiment 1 subjective ratings rmANOVA | | | | |
| --- | --- | --- | --- | --- | --- |
| **Experiment 1** | | | | | |
| **Effect** | **DFn** | **DFd** | **F** | **p[GG]** | **partial η^2^** |
| **valence** |  |  |  |  |  |
| time | 2 | 42 | 4.488 | **0.021** | 0.176 |
| condition | 1 | 21 | 18.297 | **< .001** | 0.466 |
| time x condition | 2 | 42 | 20.077 | **< .001** | 0.489 |
| **arousal** |  |  |  |  |  |
| time | 2 | 40 | 0.57 | 0.552 | 0.028 |
| condition | 1 | 20 | 4.633 | **0.044** | 0.188 |
| time x condition | 2 | 40 | 11.764 | **< .001** | 0.37 |
| **anxiety** |  |  |  |  |  |
| time | 2 | 42 | 10.355 | **< .001** | 0.33 |
| condition | 1 | 21 | 23.063 | **< .001** | 0.523 |
| time x condition | 2 | 42 | 20.764 | **< .001** | 0.497 |
| **US expectancy** |  |  |  |  |  |
| time | 1 | 22 | 8.313 | **0.009** | 0.274 |
| condition | 1 | 22 | 74.143 | **< .001** | 0.771 |
| time x condition | 1 | 22 | 15.825 | **< .001** | 0.418 |
| **CS-US contingency** | | | | | |
| time | 2 | 44 | 52.046 | **< .001** | 0.703 |
| condition | 1 | 22 | 131.989 | **< .001** | 0.857 |
| time x condition | 2 | 44 | 45.051 | **< .001** | 0.672 |

* condition contains CTX+, CTX-, ITI

| Table S6 | post-hoc t-test CTX+ v.s. CTX- | | | |
| --- | --- | --- | --- | --- |
| **Experiment 1** | | | | |
| **rating time** | **df** | **t** | **p** | **hedge's g** |
| **valence** |  |  |  |  |
| pre-acq | 21 | -0.19 | 0.85 | -0.04 |
| post-acq | 22 | -6.12 | **< .001** | -1.23 |
| post-recall | 22 | -0.05 | 0.96 | -0.01 |
| **arousal** |  |  |  |  |
| pre-acq | 20 | -1.33 | 0.20 | -0.28 |
| post-acq | 22 | 4.17 | **< .001** | 0.84 |
| post-recall | 22 | 1.15 | .26 | 0.23 |
| **anxiety** |  |  |  |  |
| pre-acq | 21 | -1.21 | 0.24 | -0.25 |
| post-acq | 22 | 6.74 | **< .001** | 1.36 |
| post-recall | 22 | 2.49 | **0.021** | 0.50 |
| **US expectancy** |  |  |  |  |
| post-acq | 22 | 13.21 | **< .001** | 2.66 |
| post-recall | 22 | 4.16 | **< .001** | 0.84 |
| **CS-US contingency** |  |  |  |  |
| post-acq | 22 | 11.96 | **< .001** | 2.41 |
| post-recall | 22 | 1.68 | 0.11 | 0.34 |
| post-recall recall acq | 22 | 8.69 | **< .001** | 1.75 |

| Table S7 | Experiment 2 subjective ratings rmANOVA | | | | |
| --- | --- | --- | --- | --- | --- |
| **Experiment 2** | | | | | |
| **Effect** | **DFn** | **DFd** | **F** | **p[GG]** | **partial η^2^** |
| **valence** |  |  |  |  |  |
| time | 2 | 54 | 5.893 | **0.005** | 0.179 |
| condition | 1 | 27 | 6.584 | **0.016** | 0.196 |
| time x condition | 2 | 54 | 7.792 | **0.002** | 0.224 |
| **arousal** |  |  |  |  |  |
| time | 2 | 54 | 0.473 | 0.609 | 0.017 |
| condition | 1 | 27 | 8.176 | **0.008** | 0.232 |
| time x condition | 2 | 54 | 5.224 | **0.009** | 0.162 |
| **anxiety** |  |  |  |  |  |
| time | 2 | 54 | 7.408 | **0.003** | 0.215 |
| condition | 1 | 27 | 7.497 | **0.011** | 0.217 |
| time x condition | 2 | 54 | 5.335 | **0.015** | 0.165 |
| **US expectancy** |  |  |  |  |  |
| time | 1 | 27 | 16.556 | **< .001** | 0.38 |
| condition | 1 | 27 | 20.215 | **< .001** | 0.428 |
| time x condition | 1 | 27 | 9.655 | **0.004** | 0.263 |
| **CS-US contingency** | | | | | |
| time | 2 | 54 | 73.237 | **< .001** | 0.731 |
| condition | 1 | 27 | 23.029 | **< .001** | 0.46 |
| time x condition | 2 | 54 | 15.592 | **< .001** | 0.366 |

* condition contains CTX+, CTX-, ITI

| Table S8 | post-hoc t-test CTX+ v.s. CTX- | | | |
| --- | --- | --- | --- | --- |
| **Experiment 2** | | | | |
| **rating time** | **df** | **t** | **p** | **hedge's g** |
| **valence** |  |  |  |  |
| pre-acq | 27 | 0.82 | 0.42 | 0.15 |
| post-acq | 27 | -3.61 | **0.001** | -0.66 |
| post-recall | 27 | -0.75 | 0.46 | -0.14 |
| **arousal** |  |  |  |  |
| pre-acq | 27 | -1.22 | 0.23 | -0.22 |
| post-acq | 27 | 3.51 | **0.002** | 0.64 |
| post-recall | 27 | 1.56 | 0.13 | 0.29 |
| **anxiety** |  |  |  |  |
| pre-acq | 27 | -0.10 | 0.92 | -0.02 |
| post-acq | 27 | 3.06 | **0.005** | 0.56 |
| post-recall | 27 | 1.67 | 0.11 | 0.31 |
| **US expectancy** |  |  |  |  |
| post-acq | 27 | 5.05 | **< .001** | 0.93 |
| post-recall | 27 | 2.43 | **0.022** | 0.45 |
| **CS-US contingency** |  |  |  |  |
| post-acq | 27 | 4.61 | **< .001** | 0.85 |
| post-recall | 27 | 0.61 | 0.55 | 0.11 |
| post-recall recall acq | 27 | 4.50 | **< .001** | 0.83 |

| Table S9 |  |  |  |
| --- | --- | --- | --- |
| Experiment 1 SEBR LME omnibus effects | | | |
| **Predictors** | **df** | **F** | **p** |
| condition | 2, 1227.2 | 7.92 | **<0.001** |
| probe_index | 1, 1227.3 | 40.51 | **<0.001** |
| probe_index_trial | 1, 1227.2 | 3.97 | **0.047** |
| condition x probe_index | 2, 1227.2 | 3.78 | **0.023** |

| Experiment 2 SEBR LME omnibus effects | | | |
| --- | --- | --- | --- |
| **Predictors** | **df** | **F** | **p** |
| condition | 2, 1514.6 | 3.11 | **0.045** |
| probe_index | 1, 1514.5 | 46.3 | **<0.001** |
| probe_index_trial | 1, 1514.4 | 3.29 | 0.070 |
| condition x probe_index | 2, 1514.5 | 0.38 | 0.683 |

| Experiment 1 SEBR LME summary table | | | |
| --- | --- | --- | --- |
| **Predictors** | **df** | **t** | **p** |
| CTX- | 1246 | -0.12 | 0.904 |
| ITI | 1246 | -3.47 | **0.001** |
| probe_index | 1246 | -4.31 | **<0.001** |
| ITI x probe_index | 1246 | 2.23 | **0.026** |

| Experiment 2 SEBR LME summary table | | | |
| --- | --- | --- | --- |
| **Predictors** | **df** | **t** | **p** |
| CTX- | 1539 | -0.02 | 0.984 |
| ITI | 1539 | -2.14 | **0.032** |
| probe_index | 1539 | -4.66 | **<0.001** |
| ITI x probe_index | 1539 | 0.77 | 0.443 |

* condition contains CTX+, CTX-, ITI; probe_index represents probe number over time (i.e., 1 to 58); probe_index_trial represents the order of probes delivered in each trial (i.e., the first or the second probe)

| Table S10 | rmANOVA of Experiment 3 and 4 verval reports | | | | |  |  |  |  |  |  |
| --- | --- | --- | --- | --- | --- | --- | --- | --- | --- | --- | --- |
|  |  |  | **Experiment 3** | |  |  |  |  | **Experiment 4** | |  |
| **Effect** | **DFn** | **DFd** | **F** | **p[GG]** | **partial η^2^** | **Effect** | **DFn** | **DFd** | **F** | **p[GG]** | **partial η^2^** |
| **valence** |  |  |  |  |  | **valence** |  |  |  |  |  |
| trial | 1 | 28 | 1.510 | 0.229 | 0.051 | trial | 1 | 23 | 0.252 | 0.621 | 0.011 |
| CON | 4 | 112 | 33.340 | **<0.001** | 0.544 | CON | 4 | 92 | 12.932 | **<0.001** | 0.360 |
| trial x CON | 4 | 112 | 10.201 | **<0.001** | 0.267 | trial x CON | 4 | 92 | 5.029 | **0.004** | 0.179 |
| **arousal** |  |  |  |  |  | **arousal** |  |  |  |  |  |
| trial | 1 | 28 | 26.441 | **<0.001** | 0.486 | trial | 1 | 23 | 2.883 | 0.103 | 0.111 |
| CON | 4 | 112 | 76.252 | **<0.001** | 0.731 | CON | 4 | 92 | 33.250 | **<0.001** | 0.591 |
| trial x CON | 4 | 112 | 8.457 | **<0.001** | 0.232 | trial x CON | 4 | 92 | 7.930 | **<0.001** | 0.256 |
| **CS-US contingency** | |  |  |  |  | **CS-US contingency** | |  |  |  |  |
| trial | 2 | 56 | 24.539 | **<0.001** | 0.467 | trial | 2 | 46 | 25.302 | **<0.001** | 0.524 |
| CON | 4 | 112 | 141.890 | **<0.001** | 0.835 | CON | 4 | 92 | 82.764 | **<0.001** | 0.783 |
| trial x CON | 8 | 224 | 68.117 | **<0.001** | 0.709 | trial x CON | 8 | 184 | 38.479 | **<0.001** | 0.626 |

* condition contains CON_A_+, CON_A_-, CON_A_-ER, CON_B_-, CON_B_-EA; linear trial index represents time

| Table S11 | Experiment 3 and 4 acquisition fixed SCR and pupil dilation paired t-test effect sizes | | | | | | | | | |
| --- | --- | --- | --- | --- | --- | --- | --- | --- | --- | --- |
| **Experiment 3 Experiment 4** | | | | | | | | | | |
|  | **df** | **t** | **p** | **d** | **g df t p d g** | | | | | |
| **fixed-latency SCR to CTX onset** | | | | | | | | | | |
| CON_A_+/CON_A_- | 27 | 3.027 | **0.005** | 0.572 | 0.556 | 22 | 3.692 | **0.001** | 0.770 | 0.743 |
| CON_A_+/CON_A_-ER | 27 | 2.800 | **0.009** | 0.529 | 0.514 | 22 | 3.814 | **<0.001** | 0.795 | 0.768 |
| CON_A_+/CON_B_-EA | 27 | 2.909 | **0.007** | 0.550 | 0.534 | 22 | 3.150 | **0.005** | 0.657 | 0.634 |
| CON_A_+/CON_B_- | 27 | 3.764 | **<0.001** | 0.711 | 0.691 | 22 | 3.527 | **0.002** | 0.735 | 0.710 |
| CON_A_+/CTX- | 27 | 3.270 | **0.003** | 0.618 | 0.601 | 22 | 3.626 | **0.001** | 0.756 | 0.730 |
| **pupil dilation** |  |  |  |  |  | | | | | |
| CON_A_+/CON_A_- | 28 | 2.913 | **0.007** | 0.541 | 0.526 | 23 | 4.611 | **<0.001** | 0.941 | 0.910 |
| CON_A_+/CON_A_-ER | 28 | 3.285 | **0.003** | 0.610 | 0.593 | 23 | 5.033 | **<0.001** | 1.027 | 0.993 |
| CON_A_+/CON_B_-EA | 28 | 5.586 | **<0.001** | 1.037 | 1.009 | 23 | 5.167 | **<0.001** | 1.055 | 1.020 |
| CON_A_+/CON_B_- | 28 | 4.804 | **<0.001** | 0.892 | 0.868 | 23 | 4.721 | **<0.001** | 0.964 | 0.932 |
| CON_A_+/CTX- | 28 | 4.395 | **<0.001** | 0.816 | 0.794 | 23 | 5.331 | **<0.001** | 1.088 | 1.052 |
| * only non-reinforced trials were included in analysis | | | | |  | | | | | |
| p-values were not Holm-Bonferroni corrected | | | | |  | | | | | |

| Table S12 | Experiment 3 and 4 extinction SEBR LME summary table | | | | | |
| --- | --- | --- | --- | --- | --- | --- |
| **Experiment 3 Experiment 4** | | | | | | |
| **Predictors** | **df** | **t** | **p** | **df** | **t** | **p** |
| trial | 2319 | -14.01 | **<0.001** | 2064 | -13.98 | **<0.001** |
| CON_A_- | 2319 | -3.78 | **<0.001** | 2064 | -3.78 | **<0.001** |
| CON_A_-ER | 2319 | -3.39 | **0.001** | 2064 | -5.23 | **<0.001** |
| CON_B_- | 2319 | -2.67 | **0.008** | 2064 | -4.11 | **<0.001** |
| CON_B_-EA | 2319 | -3.11 | **0.002** | 2064 | -4.01 | **<0.001** |
| trial x CON_A_- | 2319 | 2.14 | **0.032** | 2064 | 1.85 | 0.064 |
| trial x CON_A_-ER | 2319 | 1.35 | 0.18 | 2064 | 2.71 | **0.007** |
| trial x CON_B_- | 2319 | 0.8 | 0.42 | 2064 | 2.15 | **0.031** |
| trial x CON_B_-EA | 2319 | 1.44 | 0.15 | 2064 | 2.18 | **0.029** |

| Table S13 | Experiment 3 extinction SEBR paired t-test effect sizes | | | | | | | |  |  |
| --- | --- | --- | --- | --- | --- | --- | --- | --- | --- | --- |
| **CON_A_+ vs. CON_A_-** | | | |  |  | **CON_A_+ vs. CON_A_-ER** | | | |  |
|  | **df** | **t** | **p** | **d** | **g** | **df** | **t** | **p** | **d** | **g** |
| first 1 | 25 | 2.889 | **0.008** | 0.556 | 0.540 | 24 | 2.264 | **0.033** | 0.436 | 0.423 |
| first 2 | 26 | 3.118 | **0.004** | 0.600 | 0.583 | 26 | 2.406 | **0.024** | 0.463 | 0.449 |
| first 3 | 26 | 2.700 | **0.012** | 0.520 | 0.504 | 26 | 1.898 | 0.069 | 0.365 | 0.355 |
| first 4 | 26 | 1.610 | 0.120 | 0.310 | 0.301 | 26 | 1.356 | 0.187 | 0.261 | 0.253 |
| first 5 | 26 | 2.058 | 0.050 | 0.396 | 0.385 | 26 | 1.602 | 0.121 | 0.308 | 0.299 |
| first 6 | 26 | 2.207 | **0.036** | 0.425 | 0.412 | 26 | 1.743 | 0.093 | 0.335 | 0.326 |
| first 7 | 26 | 1.965 | 0.060 | 0.378 | 0.367 | 26 | 1.556 | 0.132 | 0.299 | 0.291 |
| first 8 | 26 | 1.650 | 0.111 | 0.318 | 0.308 | 26 | 1.435 | 0.163 | 0.276 | 0.268 |
| first 9 | 26 | 1.659 | 0.109 | 0.319 | 0.310 | 26 | 1.582 | 0.126 | 0.305 | 0.296 |
| first 10 | 26 | 1.716 | 0.098 | 0.330 | 0.321 | 26 | 1.689 | 0.103 | 0.325 | 0.316 |
| first 11 | 26 | 1.783 | 0.086 | 0.343 | 0.333 | 26 | 1.756 | 0.091 | 0.338 | 0.328 |
| first 12 | 26 | 1.861 | 0.074 | 0.358 | 0.348 | 26 | 1.845 | 0.076 | 0.355 | 0.345 |
| first 13 | 26 | 2.019 | 0.054 | 0.389 | 0.377 | 26 | 1.919 | 0.066 | 0.369 | 0.358 |
| first 14 | 26 | 2.205 | **0.037** | 0.424 | 0.412 | 26 | 2.069 | **0.049** | 0.398 | 0.387 |
| first 15 | 26 | 2.239 | **0.034** | 0.431 | 0.418 | 26 | 2.108 | **0.045** | 0.406 | 0.394 |
| first 16 | 26 | 2.053 | 0.050 | 0.395 | 0.383 | 26 | 2.139 | **0.042** | 0.412 | 0.400 |

| **CON_A_+ vs. CON_B_-EA CON_A_+ vs. CON_B_- CON_A_+ vs. CTX-** | | | | | | | | | | | | | | | |
| --- | --- | --- | --- | --- | --- | --- | --- | --- | --- | --- | --- | --- | --- | --- | --- |
| **df** | | **t** | **p** | **d** | **g** | **df** | **t** | **p** | **d** | **g** | **df** | **t** | **p** | **d** | **g** |
| first 1 | 24 | 1.640 | 0.114 | 0.316 | 0.306 | 25 | 2.869 | **0.008** | 0.552 | 0.536 | 25 | 3.004 | **0.006** | 0.578 | 0.561 |
| first 2 | 26 | 2.172 | **0.039** | 0.418 | 0.406 | 26 | 2.034 | 0.052 | 0.391 | 0.380 | 26 | 2.791 | **0.010** | 0.537 | 0.521 |
| first 3 | 26 | 1.388 | 0.177 | 0.267 | 0.259 | 26 | 1.405 | 0.172 | 0.270 | 0.263 | 26 | 1.984 | 0.058 | 0.382 | 0.371 |
| first 4 | 26 | 1.051 | 0.303 | 0.202 | 0.196 | 26 | 0.888 | 0.383 | 0.171 | 0.166 | 26 | 1.292 | 0.208 | 0.249 | 0.241 |
| first 5 | 26 | 1.639 | 0.113 | 0.315 | 0.306 | 26 | 1.325 | 0.197 | 0.255 | 0.248 | 26 | 1.704 | 0.100 | 0.328 | 0.318 |
| first 6 | 26 | 1.849 | 0.076 | 0.356 | 0.346 | 26 | 1.495 | 0.147 | 0.288 | 0.279 | 26 | 1.855 | 0.075 | 0.357 | 0.347 |
| first 7 | 26 | 1.704 | 0.100 | 0.328 | 0.318 | 26 | 1.417 | 0.168 | 0.273 | 0.265 | 26 | 1.689 | 0.103 | 0.325 | 0.316 |
| first 8 | 26 | 1.512 | 0.142 | 0.291 | 0.283 | 26 | 1.240 | 0.226 | 0.239 | 0.232 | 26 | 1.489 | 0.148 | 0.287 | 0.278 |
| first 9 | 26 | 1.518 | 0.141 | 0.292 | 0.284 | 26 | 1.401 | 0.173 | 0.270 | 0.262 | 26 | 1.576 | 0.127 | 0.303 | 0.295 |
| first 10 | 26 | 1.603 | 0.121 | 0.309 | 0.300 | 26 | 1.434 | 0.163 | 0.276 | 0.268 | 26 | 1.649 | 0.111 | 0.317 | 0.308 |
| first 11 | 26 | 1.468 | 0.154 | 0.282 | 0.274 | 26 | 1.492 | 0.148 | 0.287 | 0.279 | 26 | 1.671 | 0.107 | 0.322 | 0.312 |
| first 12 | 26 | 1.454 | 0.158 | 0.280 | 0.272 | 26 | 1.535 | 0.137 | 0.295 | 0.287 | 26 | 1.729 | 0.096 | 0.333 | 0.323 |
| first 13 | 26 | 1.540 | 0.136 | 0.296 | 0.288 | 26 | 1.744 | 0.093 | 0.336 | 0.326 | 26 | 1.865 | 0.073 | 0.359 | 0.349 |
| first 14 | 26 | 1.680 | 0.105 | 0.323 | 0.314 | 26 | 1.864 | 0.074 | 0.359 | 0.348 | 26 | 2.022 | 0.054 | 0.389 | 0.378 |
| first 15 | 26 | 1.775 | 0.088 | 0.342 | 0.332 | 26 | 1.926 | 0.065 | 0.371 | 0.360 | 26 | 2.094 | **0.046** | 0.403 | 0.391 |
| first 16 | 26 | 1.688 | 0.103 | 0.325 | 0.315 | 26 | 1.824 | 0.080 | 0.351 | 0.341 | 26 | 2.016 | 0.054 | 0.388 | 0.377 |

| * first N means average over the first N subsets; d: cohen's d; g: hedge's g |
| --- |
| p-values were not Holm-Bonferroni corrected (5 comparisons for each summarisation over subsets) |

| Table S14 | | Experiment 4 extinction SEBR paired t-test effect sizes | | | | | | |  |  |
| --- | --- | --- | --- | --- | --- | --- | --- | --- | --- | --- |
|  | **CON_A_+ vs. CON_A_-** | | |  |  | **CON_A_+ vs. CON_A_-ER** | | |  |  |
|  | **df** | **t** | **p** | **d** | **g** | **df** | **t** | **p** | **d** | **g** |
| first 1 | 22 | 2.777 | **0.011** | 0.567 | 0.548 | 22 | 3.064 | **0.006** | 0.625 | 0.605 |
| first 2 | 23 | 2.485 | **0.021** | 0.507 | 0.491 | 23 | 3.719 | **0.001** | 0.759 | 0.734 |
| first 3 | 23 | 2.200 | **0.038** | 0.449 | 0.434 | 23 | 3.430 | **0.002** | 0.700 | 0.677 |
| first 4 | 23 | 2.183 | **0.039** | 0.446 | 0.431 | 23 | 3.002 | **0.006** | 0.613 | 0.593 |
| first 5 | 23 | 2.969 | **0.007** | 0.606 | 0.586 | 23 | 3.602 | **0.002** | 0.735 | 0.711 |
| first 6 | 23 | 3.726 | **0.001** | 0.760 | 0.735 | 23 | 4.135 | **<0.001** | 0.844 | 0.816 |
| first 7 | 23 | 4.055 | **<0.001** | 0.828 | 0.800 | 23 | 3.915 | **<0.001** | 0.799 | 0.773 |
| first 8 | 23 | 3.561 | **0.002** | 0.727 | 0.703 | 23 | 3.865 | **<0.001** | 0.789 | 0.763 |
| first 9 | 23 | 3.614 | **0.001** | 0.738 | 0.713 | 23 | 4.462 | **<0.001** | 0.911 | 0.881 |
| first 10 | 23 | 3.871 | **<0.001** | 0.790 | 0.764 | 23 | 4.591 | **<0.001** | 0.937 | 0.906 |
| first 11 | 23 | 3.932 | **<0.001** | 0.803 | 0.776 | 23 | 4.603 | **<0.001** | 0.939 | 0.908 |
| first 12 | 23 | 3.978 | **<0.001** | 0.812 | 0.785 | 23 | 4.385 | **<0.001** | 0.895 | 0.865 |
| first 13 | 23 | 4.067 | **<0.001** | 0.830 | 0.803 | 23 | 4.595 | **<0.001** | 0.938 | 0.907 |
| first 14 | 23 | 4.461 | **<0.001** | 0.911 | 0.880 | 23 | 5.156 | **<0.001** | 1.052 | 1.018 |
| first 15 | 23 | 4.277 | **<0.001** | 0.873 | 0.844 | 23 | 5.148 | **<0.001** | 1.051 | 1.016 |
| first 16 | 23 | 3.911 | **<0.001** | 0.798 | 0.772 | 23 | 5.167 | **<0.001** | 1.055 | 1.020 |

| **CON_A_+ vs. CON_B_-EA CON_A_+ vs. CON_B_- CON_A_+ vs. CTX-** | | | | | | | | | | | | | | | |
| --- | --- | --- | --- | --- | --- | --- | --- | --- | --- | --- | --- | --- | --- | --- | --- |
|  | **df** | **t** | **p** | **d** | **g** | **df** | **t** | **p** | **d** | **g** | **df** | **t** | **p** | **d** | **g** |
| first 1 | 23 | 2.998 | **0.006** | 0.612 | 0.592 | 22 | 2.620 | **0.016** | 0.535 | 0.517 | 23 | 3.478 | **0.002** | 0.710 | 0.687 |
| first 2 | 23 | 2.061 | 0.051 | 0.421 | 0.407 | 23 | 2.467 | **0.022** | 0.504 | 0.487 | 23 | 2.878 | **0.008** | 0.588 | 0.568 |
| first 3 | 23 | 1.652 | 0.112 | 0.337 | 0.326 | 23 | 1.858 | 0.076 | 0.379 | 0.367 | 23 | 2.814 | **0.010** | 0.574 | 0.555 |
| first 4 | 23 | 1.403 | 0.174 | 0.286 | 0.277 | 23 | 1.949 | 0.064 | 0.398 | 0.385 | 23 | 2.417 | **0.024** | 0.493 | 0.477 |
| first 5 | 23 | 2.233 | **0.036** | 0.456 | 0.441 | 23 | 2.468 | **0.021** | 0.504 | 0.487 | 23 | 3.339 | **0.003** | 0.682 | 0.659 |
| first 6 | 23 | 2.441 | **0.023** | 0.498 | 0.482 | 23 | 3.121 | **0.005** | 0.637 | 0.616 | 23 | 4.011 | **<0.001** | 0.819 | 0.792 |
| first 7 | 23 | 2.571 | **0.017** | 0.525 | 0.507 | 23 | 2.903 | **0.008** | 0.593 | 0.573 | 23 | 4.092 | **<0.001** | 0.835 | 0.808 |
| first 8 | 23 | 2.440 | **0.023** | 0.498 | 0.482 | 23 | 2.721 | **0.012** | 0.555 | 0.537 | 23 | 3.801 | **<0.001** | 0.776 | 0.750 |
| first 9 | 23 | 2.994 | **0.006** | 0.611 | 0.591 | 23 | 3.280 | **0.003** | 0.670 | 0.647 | 23 | 4.396 | **<0.001** | 0.897 | 0.868 |
| first 10 | 23 | 3.326 | **0.003** | 0.679 | 0.657 | 23 | 3.641 | **0.001** | 0.743 | 0.719 | 23 | 4.727 | **<0.001** | 0.965 | 0.933 |
| first 11 | 23 | 3.292 | **0.003** | 0.672 | 0.650 | 23 | 3.199 | **0.004** | 0.653 | 0.631 | 23 | 4.563 | **<0.001** | 0.931 | 0.901 |
| first 12 | 23 | 3.121 | **0.005** | 0.637 | 0.616 | 23 | 3.051 | **0.006** | 0.623 | 0.602 | 23 | 4.448 | **<0.001** | 0.908 | 0.878 |
| first 13 | 23 | 2.954 | **0.007** | 0.603 | 0.583 | 23 | 3.216 | **0.004** | 0.657 | 0.635 | 23 | 4.520 | **<0.001** | 0.923 | 0.892 |
| first 14 | 23 | 3.336 | **0.003** | 0.681 | 0.658 | 23 | 3.481 | **0.002** | 0.711 | 0.687 | 23 | 4.939 | **<0.001** | 1.008 | 0.975 |
| first 15 | 23 | 2.811 | **0.010** | 0.574 | 0.555 | 23 | 3.265 | **0.003** | 0.666 | 0.644 | 23 | 4.567 | **<0.001** | 0.932 | 0.901 |
| first 16 | 23 | 2.748 | **0.011** | 0.561 | 0.542 23 3.222 **0.004** 0.658 0.636 23 4.441 **<0.001** 0.906 0.877 | | | | | | | | | | |

| * first N means average over the first N subsets; d: cohen's d; g: hedge's g |
| --- |
| p-values were not Holm-Bonferroni corrected (5 comparisons for each summarisation over subsets) |

| Table S15 |  |  |  |  |  |  |
| --- | --- | --- | --- | --- | --- | --- |
| Experiment 3 and 4 extinction, LME omnibus effect of pupil dilation | | | | | | |
|  | **Experiment 3** | |  | **Experiment 4** | |  |
| **Predictors** | **df** | **F** | **p** | **df** | **F** | **p** |
| trial | 1, 2491.0 | 85.25 | **< 0.001** | 1, 2070.0 | 42.46 | **< 0.001** |
| condition | 4, 2490.3 | 5.88 | **< 0.001** | 4, 2070.2 | 4.55 | **0.001** |
| trial x condition | 4, 2490.5 | 0.32 | 0.84 | 4, 2070.3 | 1.15 | 0.33 |

| Experiment 3 and 4 extinction, LME summary table of pupil dilation | | | | | | |
| --- | --- | --- | --- | --- | --- | --- |
|  | **Experiment 3** | |  | **Experiment 4** | |  |
| **Predictors** | **df** | **t** | **p** | **df** | **t** | **p** |
| trial | 2516 | -5.69 | **< 0.001** | 2091 | -4.50 | **< 0.001** |
| CON_A_- | 2516 | -0.57 | 0.57 | 2091 | -0.78 | 0.43 |
| CON_A_-ER | 2516 | -1.72 | 0.085 | 2091 | -0.34 | 0.73 |
| CON_B_- | 2516 | -2.29 | **0.022** | 2091 | -2.66 | **0.008** |
| CON_B_-EA | 2516 | -4.53 | **< 0.001** | 2091 | -3.59 | **< 0.001** |

| Table S16 |  | Experiment 3 extinction pupil dilation paired t-test effect sizes | | | | | | | |  |
| --- | --- | --- | --- | --- | --- | --- | --- | --- | --- | --- |
| **CON_A_+ vs. CON_A_-** | | | |  |  | **CON_A_+ vs. CON_A_-ER** | | |  |  |
| **df** | | **t** | **p** | **d** | **g** | **df** | **t** | **p** | **d** | **g** |
| first 1 | 28 | 3.079 | **0.005** | 0.572 | 0.556 | 28 | 2.839 | **0.008** | 0.527 | 0.513 |
| first 2 | 28 | 3.464 | **0.002** | 0.643 | 0.626 | 28 | 2.906 | **0.007** | 0.540 | 0.525 |
| first 3 | 28 | 1.123 | 0.271 | 0.209 | 0.203 | 28 | 1.808 | 0.081 | 0.336 | 0.327 |
| first 4 | 28 | -0.130 | 0.897 | -0.024 | -0.024 | 28 | 1.267 | 0.216 | 0.235 | 0.229 |
| first 5 | 28 | 0.078 | 0.939 | 0.014 | 0.014 | 28 | 1.247 | 0.223 | 0.232 | 0.225 |
| first 6 | 28 | 0.510 | 0.614 | 0.095 | 0.092 | 28 | 1.369 | 0.182 | 0.254 | 0.247 |
| first 7 | 28 | 0.231 | 0.819 | 0.043 | 0.042 | 28 | 1.208 | 0.237 | 0.224 | 0.218 |
| first 8 | 28 | -0.088 | 0.931 | -0.016 | -0.016 | 28 | 1.068 | 0.295 | 0.198 | 0.193 |
| first 9 | 28 | -0.223 | 0.825 | -0.041 | -0.040 | 28 | 0.739 | 0.466 | 0.137 | 0.133 |
| first 10 | 28 | -0.167 | 0.868 | -0.031 | -0.030 | 28 | 0.811 | 0.424 | 0.151 | 0.147 |
| first 11 | 28 | 0.218 | 0.829 | 0.041 | 0.039 | 28 | 1.324 | 0.196 | 0.246 | 0.239 |
| first 12 | 28 | 0.330 | 0.744 | 0.061 | 0.060 | 28 | 1.513 | 0.141 | 0.281 | 0.273 |
| first 13 | 28 | 0.340 | 0.736 | 0.063 | 0.061 | 28 | 1.437 | 0.162 | 0.267 | 0.260 |
| first 14 | 28 | 0.454 | 0.653 | 0.084 | 0.082 | 28 | 1.614 | 0.118 | 0.300 | 0.292 |
| first 15 | 28 | 0.362 | 0.720 | 0.067 | 0.065 | 28 | 2.032 | 0.052 | 0.377 | 0.367 |
| first 16 | 28 | 0.081 | 0.936 | 0.015 | 0.015 | 28 | 1.467 | 0.154 | 0.272 | 0.265 |

| **CON_A_+ vs. CON_B_-EA CON_A_+ vs. CON_B_- CON_A_+ vs. CTX-** | | | | | | | | | | | | | | | |
| --- | --- | --- | --- | --- | --- | --- | --- | --- | --- | --- | --- | --- | --- | --- | --- |
| **df** | | **t** | **p** | **d** | **g** | **df** | **t** | **p** | **d** | **g** | **df** | **t** | **p** | **d** | **g** |
| first 1 | 28 | 5.784 | **< 0.001** | 1.074 | 1.045 | 28 | 2.993 | **0.006** | 0.556 | 0.541 | 28 | 4.943 | **< 0.001** | 0.918 | 0.893 |
| first 2 | 28 | 6.367 | **< 0.001** | 1.182 | 1.150 | 28 | 4.756 | **< 0.001** | 0.883 | 0.859 | 28 | 5.498 | **< 0.001** | 1.021 | 0.993 |
| first 3 | 28 | 4.718 | **< 0.001** | 0.876 | 0.852 | 28 | 2.564 | **0.016** | 0.476 | 0.463 | 28 | 2.969 | **0.006** | 0.551 | 0.536 |
| first 4 | 28 | 3.718 | **< 0.001** | 0.690 | 0.672 | 28 | 1.364 | 0.184 | 0.253 | 0.246 | 28 | 1.751 | 0.091 | 0.325 | 0.316 |
| first 5 | 28 | 4.729 | **< 0.001** | 0.878 | 0.854 | 28 | 1.900 | 0.068 | 0.353 | 0.343 | 28 | 2.147 | **0.041** | 0.399 | 0.388 |
| first 6 | 28 | 5.208 | **< 0.001** | 0.967 | 0.941 | 28 | 2.611 | **0.014** | 0.485 | 0.472 | 28 | 2.679 | **0.012** | 0.497 | 0.484 |
| first 7 | 28 | 4.817 | **< 0.001** | 0.894 | 0.870 | 28 | 1.961 | 0.060 | 0.364 | 0.354 | 28 | 2.271 | **0.031** | 0.422 | 0.410 |
| first 8 | 28 | 4.092 | **< 0.001** | 0.760 | 0.739 | 28 | 1.652 | 0.110 | 0.307 | 0.298 | 28 | 1.972 | 0.059 | 0.366 | 0.356 |
| first 9 | 28 | 4.999 | **< 0.001** | 0.928 | 0.903 | 28 | 1.782 | 0.086 | 0.331 | 0.322 | 28 | 2.135 | **0.042** | 0.396 | 0.386 |
| first 10 | 28 | 5.386 | **< 0.001** | 1.000 | 0.973 | 28 | 1.875 | 0.071 | 0.348 | 0.339 | 28 | 2.266 | **0.031** | 0.421 | 0.409 |
| first 11 | 28 | 5.770 | **< 0.001** | 1.071 | 1.042 | 28 | 2.187 | **0.037** | 0.406 | 0.395 | 28 | 2.820 | **0.009** | 0.524 | 0.510 |
| first 12 | 28 | 6.979 | **< 0.001** | 1.296 | 1.261 | 28 | 2.686 | **0.012** | 0.499 | 0.485 | 28 | 3.398 | **0.002** | 0.631 | 0.614 |
| first 13 | 28 | 7.136 | **< 0.001** | 1.325 | 1.289 | 28 | 2.876 | **0.008** | 0.534 | 0.520 | 28 | 3.609 | **0.001** | 0.670 | 0.652 |
| first 14 | 28 | 6.778 | **< 0.001** | 1.259 | 1.225 | 28 | 2.873 | **0.008** | 0.533 | 0.519 | 28 | 3.694 | **< 0.001** | 0.686 | 0.667 |
| first 15 | 28 | 7.176 | **< 0.001** | 1.333 | 1.297 | 28 | 2.985 | **0.006** | 0.554 | 0.539 | 28 | 3.872 | **< 0.001** | 0.719 | 0.700 |
| first 16 28 | | 7.283 | **< 0.001** | 1.352 | 1.316 28 3.063 **0.005** 0.569 0.553 28 3.736 **< 0.001** 0.694 0.675 | | | | | | | | | | |

| * first N means average over the first N subsets; d: cohen's d; g: hedge's g |
| --- |
| p-values were not Holm-Bonferroni corrected (5 comparisons for each summarisation over subsets) |

| Table S17 | Experiment 4 extinction pupil dilation paired t-test effect sizes |
| --- | --- |

| **CON_A_+ vs. CON_A_- CON_A_+ vs. CON_A_-ER** | | | | | | | | | | |
| --- | --- | --- | --- | --- | --- | --- | --- | --- | --- | --- |
|  | **df** | **t** | **p** | **d** | **g** | **df** | **t** | **p** | **d** | **g** |
| first 1 | 23 | 2.432 | **0.023** | 0.496 | 0.480 | 23 | 3.357 | **0.003** | 0.685 | 0.663 |
| first 2 | 23 | 2.767 | **0.011** | 0.565 | 0.546 | 23 | 3.866 | **0.001** | 0.789 | 0.763 |
| first 3 | 23 | 0.544 | 0.591 | 0.111 | 0.107 | 23 | 0.669 | 0.510 | 0.137 | 0.132 |
| first 4 | 23 | 0.200 | 0.844 | 0.041 | 0.039 | 23 | -0.277 | 0.784 | -0.057 | -0.055 |
| first 5 | 23 | 0.837 | 0.411 | 0.171 | 0.165 | 23 | 0.194 | 0.848 | 0.040 | 0.038 |
| first 6 | 23 | 1.039 | 0.309 | 0.212 | 0.205 | 23 | 0.142 | 0.888 | 0.029 | 0.028 |
| first 7 | 23 | 0.737 | 0.469 | 0.150 | 0.145 | 23 | 0.016 | 0.988 | 0.003 | 0.003 |
| first 8 | 23 | 0.304 | 0.764 | 0.062 | 0.060 | 23 | 0.081 | 0.936 | 0.016 | 0.016 |
| first 9 | 23 | 0.390 | 0.700 | 0.080 | 0.077 | 23 | 0.447 | 0.659 | 0.091 | 0.088 |
| first 10 | 23 | 0.188 | 0.852 | 0.038 | 0.037 | 23 | 0.666 | 0.512 | 0.136 | 0.132 |
| first 11 | 23 | -0.016 | 0.987 | -0.003 | -0.003 | 23 | 0.731 | 0.472 | 0.149 | 0.144 |
| first 12 | 23 | -0.021 | 0.983 | -0.004 | -0.004 | 23 | 0.565 | 0.578 | 0.115 | 0.111 |
| first 13 | 23 | -0.199 | 0.844 | -0.041 | -0.039 | 23 | 0.452 | 0.656 | 0.092 | 0.089 |
| first 14 | 23 | -0.073 | 0.942 | -0.015 | -0.014 | 23 | 0.429 | 0.672 | 0.088 | 0.085 |
| first 15 | 23 | 0.073 | 0.943 | 0.015 | 0.014 | 23 | 0.692 | 0.496 | 0.141 | 0.137 |
| first 16 | 23 | -0.076 | 0.940 | -0.016 | -0.015 | 23 | 0.801 | 0.431 | 0.164 | 0.158 |

| **CON_A_+ vs. CON_B_-EA CON_A_+ vs. CON_B_- CON_A_+ vs. CTX-** | | | | | | | | | | | | | | | |
| --- | --- | --- | --- | --- | --- | --- | --- | --- | --- | --- | --- | --- | --- | --- | --- |
| **df** | | **t** | **p** | **d** | **g** | **df** | **t** | **p** | **d** | **g** | **df** | **t** | **p** | **d** | **g** |
| first 1 | 22 | 3.855 | **< 0.001** | 0.787 | 0.761 | 23 | 4.029 | **< 0.001** | 0.822 | 0.795 | 23 | 3.889 | **< 0.001** | 0.794 | 0.768 |
| first 2 | 23 | 3.892 | **< 0.001** | 0.795 | 0.768 | 23 | 5.380 | **< 0.001** | 1.098 | 1.062 | 23 | 4.461 | **< 0.001** | 0.911 | 0.880 |
| first 3 | 23 | 2.173 | **0.040** | 0.443 | 0.429 | 23 | 2.353 | **0.028** | 0.480 | 0.464 | 23 | 1.590 | 0.125 | 0.325 | 0.314 |
| first 4 | 23 | 1.880 | 0.073 | 0.384 | 0.371 | 23 | 1.731 | 0.097 | 0.353 | 0.342 | 23 | 0.899 | 0.378 | 0.184 | 0.177 |
| first 5 | 23 | 3.145 | **0.005** | 0.642 | 0.621 | 23 | 2.090 | **0.048** | 0.427 | 0.412 | 23 | 1.639 | 0.115 | 0.335 | 0.324 |
| first 6 | 23 | 3.166 | **0.004** | 0.646 | 0.625 | 23 | 2.279 | **0.032** | 0.465 | 0.450 | 23 | 1.767 | 0.091 | 0.361 | 0.349 |
| first 7 | 23 | 2.681 | **0.013** | 0.547 | 0.529 | 23 | 2.099 | **0.047** | 0.428 | 0.414 | 23 | 1.529 | 0.140 | 0.312 | 0.302 |
| first 8 | 23 | 3.040 | **0.006** | 0.621 | 0.600 | 23 | 2.181 | **0.040** | 0.445 | 0.431 | 23 | 1.590 | 0.125 | 0.325 | 0.314 |
| first 9 | 23 | 3.626 | **0.001** | 0.740 | 0.716 | 23 | 2.380 | **0.026** | 0.486 | 0.470 | 23 | 1.891 | 0.071 | 0.386 | 0.373 |
| first 10 | 23 | 3.734 | **0.001** | 0.762 | 0.737 | 23 | 2.508 | **0.020** | 0.512 | 0.495 | 23 | 1.969 | 0.061 | 0.402 | 0.389 |
| first 11 | 23 | 3.839 | **< 0.001** | 0.784 | 0.758 | 23 | 2.737 | **0.012** | 0.559 | 0.540 | 23 | 2.075 | **0.049** | 0.424 | 0.410 |
| first 12 | 23 | 3.765 | **0.001** | 0.768 | 0.743 | 23 | 2.527 | **0.019** | 0.516 | 0.499 | 23 | 1.947 | 0.064 | 0.397 | 0.384 |
| first 13 | 23 | 3.133 | **0.005** | 0.640 | 0.618 | 23 | 2.195 | **0.039** | 0.448 | 0.433 | 23 | 1.651 | 0.112 | 0.337 | 0.326 |
| first 14 | 23 | 3.255 | **0.003** | 0.664 | 0.642 | 23 | 2.271 | **0.033** | 0.464 | 0.448 | 23 | 1.819 | 0.082 | 0.371 | 0.359 |
| first 15 | 23 | 3.315 | **0.003** | 0.677 | 0.654 | 23 | 2.244 | **0.035** | 0.458 | 0.443 | 23 | 2.028 | 0.054 | 0.414 | 0.400 |
| first 16 | 23 | 3.180 | **0.004** | 0.649 | 0.628 | 23 | 2.103 | **0.047** | 0.429 | 0.415 | 23 | 1.979 | 0.060 | 0.404 | 0.391 |

| * first N means average over the first N subsets; d: cohen's d; g: hedge's g |
| --- |
| p-values were not Holm-Bonferroni corrected (5 comparisons for each summarisation over subsets) |

| Table S18 |  |  |  |  |  |  |
| --- | --- | --- | --- | --- | --- | --- |
| Experiment 3 and 4 extinction, LME omnibus effect of flexible SCR during CTX | | | | | | |
|  | **Experiment 3** | |  | **Experiment 4** | |  |
| **Predictors** | **df** | **F** | **p** | **df** | **F** | **p** |
| trial | 1, 2433.3 | 87.11 | **<0.001** | 1, 2007.7 | 196.55 | **<0.001** |
| condition | 4, 2433.0 | 4.26 | **0.002** | 4, 2007.7 | 6.41 | **<0.001** |
| trial x condition | 4, 2433.2 | 2.06 | 0.084 | 4, 2007.8 | 2.25 | 0.062 |

| Experiment 3 and 4 extinction, LME summary table of flexible SCR during CTX | | | | | | |
| --- | --- | --- | --- | --- | --- | --- |
|  | **Experiment 3** | |  | **Experiment 4** | |  |
| **Predictors** | **df** | **t** | **p** | **df** | **t** | **p** |
| trial | 2458 | -6.06 | **<0.001** | 2028 | -9.31 | **<0.001** |
| CON_A_- | 2458 | -2.54 | **0.011** | 2028 | -3.68 | **<0.001** |
| CON_A_-ER | 2458 | 0.77 | 0.442 | 2028 | -3.56 | **<0.001** |
| CON_B_- | 2458 | -1.11 | 0.266 | 2028 | -3.57 | **<0.001** |
| CON_B_-EA | 2458 | -2.74 | **0.006** | 2028 | -4.25 | **<0.001** |
| trial x CON_A_- | 2458 | 1.97 | **0.049** | 2028 | 0.97 | 0.33 |
| trial x CON_B_-EA | 2458 | 1.71 | 0.087 | 2028 | 2.97 | **0.003** |

| Table S19 |  | Experiment 3 extinction flexible SCR during CTX paired t-test effect sizes | | | |
| --- | --- | --- | --- | --- | --- |
| **CON_A_+ vs. CON_A_- CON_A_+ vs. CON_A_-ER** | | | | | |
| **df** | | **t** | **p** | **d** | **g df t p d g** |
| first 1 | 25 | -0.382 | 0.706 | -0.071 | -0.069 25 -0.819 0.420 -0.152 -0.148 |
| first 2 | 28 | 0.531 | 0.600 | 0.099 | 0.096 28 -0.719 0.478 -0.133 -0.130 |
| first 3 | 28 | 1.440 | 0.161 | 0.267 | 0.260 28 -0.288 0.775 -0.054 -0.052 |
| first 4 | 28 | 1.598 | 0.121 | 0.297 | 0.289 28 -0.007 0.994 -0.001 -0.001 |
| first 5 | 28 | 2.194 | 0.037 | 0.407 | 0.396 28 0.060 0.953 0.011 0.011 |
| first 6 | 28 | 1.959 | 0.060 | 0.364 | 0.354 28 0.058 0.954 0.011 0.010 |
| first 7 | 28 | 2.254 | **0.032** | 0.419 | 0.407 28 0.032 0.975 0.006 0.006 |
| first 8 | 28 | 2.184 | **0.037** | 0.406 | 0.395 28 -0.088 0.931 -0.016 -0.016 |
| first 9 | 28 | 2.208 | **0.036** | 0.410 | 0.399 28 -0.714 0.481 -0.133 -0.129 |
| first 10 | 28 | 1.938 | 0.063 | 0.360 | 0.350 28 -0.537 0.595 -0.100 -0.097 |
| first 11 | 28 | 1.994 | 0.056 | 0.370 | 0.360 28 -0.690 0.496 -0.128 -0.125 |
| first 12 | 28 | 1.964 | 0.060 | 0.365 | 0.355 28 -0.755 0.457 -0.140 -0.136 |
| first 13 | 28 | 2.229 | **0.034** | 0.414 | 0.403 28 -0.221 0.827 -0.041 -0.040 |
| first 14 | 28 | 2.061 | **0.049** | 0.383 | 0.372 28 -0.151 0.881 -0.028 -0.027 |
| first 15 | 28 | 1.817 | 0.080 | 0.337 | 0.328 28 -0.509 0.615 -0.094 -0.092 |
| first 16 | 28 | 1.301 | 0.204 | 0.242 | 0.235 28 -0.627 0.536 -0.116 -0.113 |

| **CON_A_+ vs. CON_B_-EA CON_A_+ vs. CON_B_- CON_A_+ vs. CTX-** | | | | | | | | | | | | | | | |
| --- | --- | --- | --- | --- | --- | --- | --- | --- | --- | --- | --- | --- | --- | --- | --- |
| **df** | | **t** | **p** | **d** | **g** | **df** | **t** | **p** | **d** | **g** | **df** | **t** | **p** | **d** | **g** |
| first 1 | 26 | 1.421 | 0.167 | 0.264 | 0.257 | 25 | -1.121 | 0.273 | -0.208 | -0.203 | 26 | -0.172 | 0.865 | -0.032 | -0.031 |
| first 2 | 28 | 2.223 | **0.034** | 0.413 | 0.402 | 28 | -0.223 | 0.825 | -0.041 | -0.040 | 28 | 0.401 | 0.692 | 0.074 | 0.072 |
| first 3 | 28 | 2.739 | **0.011** | 0.509 | 0.495 | 28 | 0.797 | 0.432 | 0.148 | 0.144 | 28 | 1.190 | 0.244 | 0.221 | 0.215 |
| first 4 | 28 | 2.686 | **0.012** | 0.499 | 0.485 | 28 | 0.953 | 0.349 | 0.177 | 0.172 | 28 | 1.349 | 0.188 | 0.250 | 0.244 |
| first 5 | 28 | 3.031 | **0.005** | 0.563 | 0.548 | 28 | 1.617 | 0.117 | 0.300 | 0.292 | 28 | 1.770 | 0.088 | 0.329 | 0.320 |
| first 6 | 28 | 2.885 | **0.007** | 0.536 | 0.521 | 28 | 1.815 | 0.080 | 0.337 | 0.328 | 28 | 1.765 | 0.088 | 0.328 | 0.319 |
| first 7 | 28 | 3.217 | **0.003** | 0.597 | 0.581 | 28 | 1.986 | 0.057 | 0.369 | 0.359 | 28 | 1.936 | 0.063 | 0.359 | 0.350 |
| first 8 | 28 | 2.875 | **0.008** | 0.534 | 0.519 | 28 | 1.652 | 0.110 | 0.307 | 0.298 | 28 | 1.691 | 0.102 | 0.314 | 0.305 |
| first 9 | 28 | 3.362 | **0.002** | 0.624 | 0.607 | 28 | 1.526 | 0.138 | 0.283 | 0.276 | 28 | 1.444 | 0.160 | 0.268 | 0.261 |
| first 10 | 28 | 3.662 | **0.001** | 0.680 | 0.662 | 28 | 1.608 | 0.119 | 0.299 | 0.291 | 28 | 1.554 | 0.131 | 0.289 | 0.281 |
| first 11 | 28 | 3.514 | **0.002** | 0.652 | 0.635 | 28 | 1.428 | 0.164 | 0.265 | 0.258 | 28 | 1.462 | 0.155 | 0.272 | 0.264 |
| first 12 | 28 | 2.961 | **0.006** | 0.550 | 0.535 | 28 | 1.132 | 0.267 | 0.210 | 0.205 | 28 | 1.256 | 0.220 | 0.233 | 0.227 |
| first 13 | 28 | 3.467 | **0.002** | 0.644 | 0.626 | 28 | 1.560 | 0.130 | 0.290 | 0.282 | 28 | 1.734 | 0.094 | 0.322 | 0.313 |
| first 14 | 28 | 3.532 | **0.001** | 0.656 | 0.638 | 28 | 1.796 | 0.083 | 0.333 | 0.324 | 28 | 1.798 | 0.083 | 0.334 | 0.325 |
| first 15 | 28 | 3.391 | **0.002** | 0.630 | 0.613 | 28 | 1.583 | 0.125 | 0.294 | 0.286 | 28 | 1.492 | 0.147 | 0.277 | 0.270 |
| first 16 | 28 | 2.865 | **0.008** | 0.532 | 0.518 | 28 | 1.486 | 0.148 | 0.276 | 0.269 | 28 | 1.170 | 0.252 | 0.217 | 0.211 |

| * first N means average over the first N subsets; d: cohen's d; g: hedge's g |
| --- |
| p-values were not Holm-Bonferroni corrected (5 comparisons for each summarisation over subsets) |

| Table S20 | Experiment 4 extinction flexible SCR during CTX paired t-test effect sizes | | | | |
| --- | --- | --- | --- | --- | --- |
| **CON_A_+ vs. CON_A_- CON_A_+ vs. CON_A_-ER** | | | | | |
| **df** | | **t** | **p** | **d** | **g df t p d g** |
| first 1 | 22 | 1.449 | 0.161 | 0.296 | 0.286 22 1.720 0.100 0.351 0.339 |
| first 2 | 22 | 2.247 | **0.035** | 0.459 | 0.444 22 1.568 0.131 0.320 0.309 |
| first 3 | 22 | 2.075 | **0.050** | 0.424 | 0.410 22 1.610 0.122 0.329 0.318 |
| first 4 | 23 | 2.265 | **0.033** | 0.462 | 0.447 23 1.728 0.097 0.353 0.341 |
| first 5 | 23 | 2.916 | **0.008** | 0.595 | 0.576 23 2.537 **0.018** 0.518 0.501 |
| first 6 | 23 | 3.540 | **0.002** | 0.723 | 0.699 23 3.032 **0.006** 0.619 0.599 |
| first 7 | 23 | 3.609 | **0.001** | 0.737 | 0.712 23 3.055 **0.006** 0.624 0.603 |
| first 8 | 23 | 3.601 | **0.002** | 0.735 | 0.711 23 3.143 **0.005** 0.642 0.620 |
| first 9 | 23 | 3.810 | **<0.001** | 0.778 | 0.752 23 3.325 **0.003** 0.679 0.656 |
| first 10 | 23 | 3.713 | **0.001** | 0.758 | 0.733 23 3.252 **0.004** 0.664 0.642 |
| first 11 | 23 | 3.626 | **0.001** | 0.740 | 0.716 23 3.195 **0.004** 0.652 0.631 |
| first 12 | 23 | 3.574 | **0.002** | 0.729 | 0.705 23 3.112 **0.005** 0.635 0.614 |
| first 13 | 23 | 3.477 | **0.002** | 0.710 | 0.686 23 3.156 **0.004** 0.644 0.623 |
| first 14 | 23 | 3.621 | **0.001** | 0.739 | 0.715 23 3.433 **0.002** 0.701 0.678 |
| first 15 | 23 | 3.500 | **0.002** | 0.714 | 0.691 23 3.352 **0.003** 0.684 0.662 |
| first 16 | 23 | 3.505 | **0.002** | 0.715 | 0.692 23 3.318 **0.003** 0.677 0.655 |

| **CON_A_+ vs. CON_B_-EA CON_A_+ vs. CON_B_- CON_A_+ vs. CTX-** | | | | | | | | | | | | | | | |
| --- | --- | --- | --- | --- | --- | --- | --- | --- | --- | --- | --- | --- | --- | --- | --- |
| **df** | | **t** | **p** | **d** | **g** | **df** | **t** | **p** | **d** | **g** | **df** | **t** | **p** | **d** | **g** |
| first 1 | 21 | 0.867 | 0.396 | 0.177 | 0.171 | 22 | 1.752 | 0.094 | 0.358 | 0.346 | 22 | 1.805 | 0.085 | 0.369 | 0.356 |
| first 2 | 22 | 1.434 | 0.166 | 0.293 | 0.283 | 22 | 1.956 | 0.063 | 0.399 | 0.386 | 22 | 2.052 | 0.052 | 0.419 | 0.405 |
| first 3 | 22 | 1.619 | 0.120 | 0.330 | 0.320 | 22 | 2.210 | **0.038** | 0.451 | 0.436 | 22 | 2.230 | **0.036** | 0.455 | 0.440 |
| first 4 | 23 | 1.777 | 0.089 | 0.363 | 0.351 | 23 | 2.884 | **0.008** | 0.589 | 0.569 | 23 | 2.461 | **0.022** | 0.502 | 0.486 |
| first 5 | 23 | 2.646 | **0.014** | 0.540 | 0.522 | 23 | 2.444 | **0.023** | 0.499 | 0.482 | 23 | 2.735 | **0.012** | 0.558 | 0.540 |
| first 6 | 23 | 3.018 | **0.006** | 0.616 | 0.596 | 23 | 2.808 | **0.010** | 0.573 | 0.554 | 23 | 3.238 | **0.004** | 0.661 | 0.639 |
| first 7 | 23 | 2.984 | **0.007** | 0.609 | 0.589 | 23 | 2.866 | **0.009** | 0.585 | 0.566 | 23 | 3.303 | **0.003** | 0.674 | 0.652 |
| first 8 | 23 | 2.635 | **0.015** | 0.538 | 0.520 | 23 | 2.943 | **0.007** | 0.601 | 0.581 | 23 | 3.248 | **0.004** | 0.663 | 0.641 |
| first 9 | 23 | 2.764 | **0.011** | 0.564 | 0.546 | 23 | 3.140 | **0.005** | 0.641 | 0.620 | 23 | 3.397 | **0.002** | 0.693 | 0.671 |
| first 10 | 23 | 2.668 | **0.014** | 0.545 | 0.527 | 23 | 3.098 | **0.005** | 0.632 | 0.611 | 23 | 3.269 | **0.003** | 0.667 | 0.645 |
| first 11 | 23 | 2.734 | **0.012** | 0.558 | 0.540 | 23 | 3.141 | **0.005** | 0.641 | 0.620 | 23 | 3.284 | **0.003** | 0.670 | 0.648 |
| first 12 | 23 | 2.671 | **0.014** | 0.545 | 0.527 | 23 | 3.054 | **0.006** | 0.623 | 0.603 | 23 | 3.201 | **0.004** | 0.653 | 0.632 |
| first 13 | 23 | 1.947 | 0.064 | 0.398 | 0.384 | 23 | 3.149 | **0.004** | 0.643 | 0.622 | 23 | 3.004 | **0.006** | 0.613 | 0.593 |
| first 14 | 23 | 2.212 | **0.037** | 0.451 | 0.437 | 23 | 3.469 | **0.002** | 0.708 | 0.685 | 23 | 3.267 | **0.003** | 0.667 | 0.645 |
| first 15 | 23 | 1.948 | 0.064 | 0.398 | 0.385 | 23 | 3.305 | **0.003** | 0.675 | 0.652 | 23 | 3.097 | **0.005** | 0.632 | 0.611 |
| first 16 | 23 | 1.632 | 0.116 | 0.333 | 0.322 | 23 | 3.207 | **0.004** | 0.655 | 0.633 | 23 | 2.957 | **0.007** | 0.604 | 0.584 |

| * first N means average over the first N subsets; d: cohen's d; g: hedge's g |
| --- |
| p-values were not Holm-Bonferroni corrected (5 comparisons for each summarisation over subsets) |
